# Genome-wide selection signatures address trait specific candidate genes in cattle indigenous to arid regions of India

**DOI:** 10.1080/10495398.2023.2290521

**Published:** 2023-12-13

**Authors:** Nidhi Sukhija, Anoop Anand Malik, Joel M. Devadasan, Aishwarya Dash, Kangabam Bidyalaxmi, D. Ravi Kumar, M. Kousalaya Devi, Anjali Choudhary, K. K. Kanaka, Rekha Sharma, Shashi Bhushan Tripathi, Saket Kumar Niranjan, Jayakumar Sivalingam, Archana Verma

**Affiliations:** a ICAR-National Dairy Research Institute, Karnal, India; b TERI School of Advanced Studies, Delhi, India; c The Energy and Resources Institute, North Eastern Regional Centre, Guwahati, India; d ICAR-National Bureau of Animal Genetic Resources, Karnal, India; e ICAR- Indian Institute of Agricultural Biotechnology, Ranchi, India

**Keywords:** ddRAD-seq, Gir, Tharparkar, selection signatures, SNPs

## Abstract

The peculiarity of Indian cattle lies in milk quality, resistance to diseases and stressors as well as adaptability. The investigation addressed selection signatures in Gir and Tharparkar cattle, belonging to arid ecotypes of India. Double digest restriction-site associated DNA sequencing (ddRAD-seq) yielded nearly 26 million high-quality reads from unrelated seven Gir and seven Tharparkar cows. In all, 19,127 high-quality SNPs were processed for selection signature analysis. An approach involving within-population composite likelihood ratio (CLR) statistics and between-population *F*_ST_ statistics was used to capture selection signatures within and between the breeds, respectively. A total of 191 selection signatures were addressed using CLR and *F*_ST_ approaches. Selection signatures overlapping 86 and 73 genes were detected as Gir- and Tharparkar-specific, respectively. Notably, genes related to production (CACNA1D, GHRHR), reproduction (ESR1, RBMS3), immunity (NOSTRIN, IL12B) and adaptation (ADAM22, ASL) were annotated to selection signatures. Gene pathway analysis revealed genes in insulin/IGF pathway for milk production, gonadotropin releasing hormone pathway for reproduction, Wnt signalling pathway and chemokine and cytokine signalling pathway for adaptation. This is the first study where selection signatures are identified using ddRAD-seq in indicine cattle breeds. The study shall help in conservation and leveraging genetic improvements in Gir and Tharparkar cattle.

## Introduction

Domestication of cattle about 8000–10,000 years ago^1^ followed by mutations, selection and demographic changes such as bottleneck effect and founder’s effect,^2^^,^^3^ cumulatively shaped the diverse breeds. During this course of evolution, zebu cattle acquired attributes of heat tolerance, longevity, low maintenance, resistance to tropical diseases and adaptability to scarcity of feed and fodder.^4^ A total of 53 cattle breeds have been registered till date in India (https://nbagr.icar.gov.in/en/registered-cattle/).^5^ Gir and Tharparkar are two of the registered zebu cattle breeds and represent the arid and semi-arid ecotypes of the Indian mainland. Gir cattle is native to Gir forests in Kathiawar region of Gujarat, while Tharparkar cattle is native to the Kachchh region of Gujarat and western parts of Rajasthan.^6^^,^^7^ The temperature in their habitat ranges from 7 to 45 °C with an average rainfall of 1000 mm.^8^ Morphologically, Gir cattle have leaf-like and the longest ears, sleepy eye appearance, typical convex head, largest hump and widest coat colour variation among all Indian cattle breeds, while Tharparkar cattle have white or light grey coat colour and convex forehead features. Performance-wise, Gir cattle produce relatively more milk (average of 2110 kg; range of 800–3300 kg) than Tharparkar (average of 1749 kg; range of 913–2147 kg),^9^ while Tharparkar displays better genetic plasticity in different climates ranging from sub-zero to fifty-degree Celsius temperature, recurrent famines and xerophytic vegetation.^10^^,^^11^

High-throughput sequencing and SNP genotyping technologies have advanced our ability to identify selection signatures in genomes, including those of cattle. These signatures, indicative of natural and artificial selection, are detected through statistical analyses of genetic data, enabling the identification of genes and mutations associated with phenotypic traits.^12^ Saravanan et al.^13^ described in detail about concepts, approaches and applications of selection signatures in livestock. The intricacies of these processes involve factors like the type of selection (hard or soft), the strength of selection and the methods used to detect these patterns in genetic data. In brief, under the influence of selection, a neutral allele gets dragged by a closely linked beneficial allele, causing hitchhiking effect. This pattern of co-inheritance left by selective sweeps decreases genetic variability in proximity, causing selection signatures on the genome^14^^,^^15^ and the phenomenon is termed as selective sweep or hitchhiking effect or genetic draft.^16–18^ They lead to reduced polymorphism, skewed site frequency spectrum and linkage disequilibrium, which can be quantified by different statistics.^19^ Composite likelihood ratio (CLR) and Wright’s fixation index (*F*_ST_) are two of them. The CLR statistic assesses the skewness in allele frequency spectra across multiple loci and factors in recombination rates to distinguish selection effects from demographic events.^20^ On the other hand, the fixation index (*F*_ST_) measures allele frequency differences between populations, with values ranging from 0, indicative of no population differentiation to 1, indicative of complete fixation difference. Elevated *F*_ST_ values at a specific locus signify positive selection, whereas low *F*_ST_ values suggest negative selection.^21^ During selection, the within-breed component of diversity fades while the between-breed component rises, measured as *F*_ST_. The greater the value of CLR and *F*_ST_, the greater will be the likelihood of genomic regions under selection.^22^

SNP markers are robust tools in population genetics to assess selection signatures,^13^ for breed classification purposes^23–25^ and for understanding various other diversity measures^3^ due to their genomic abundance and accessibility.^26^ At present, genome-wide SNP mining relies on whole genome sequencing (WGS) and SNP chips. However, SNP chips are not tailored for specific populations, leading to ascertainment bias.^27^ This limitation in chip data can be taken care of by WGS, but it is costly and involves operational difficulties. A consensus solution is genomic sub-sampling through double digest restriction-site associated DNA sequencing (ddRAD-seq) which covers roughly 5–40% of the genome.^28^^,^^29^

Erstwhile, RAD sequencing has been exercised in Sahiwal,^30^^,^^31^ Tharparkar,^32^ Badri^33^ and Vrindavani^34^ cattle breeds of India for genome-wide identification of SNPs. Though selection signature analyses have been carried out in taurine cattle breeds, there is, however, limited knowledge about selection signatures in Indian cattle breeds using ascertainment bias-free genome-wide markers. This catalysed our quest for selection signatures using ddRAD-seq in Gir and Tharparkar cattle.

## Material and methods

### Data preprocessing

**Figure 1.**
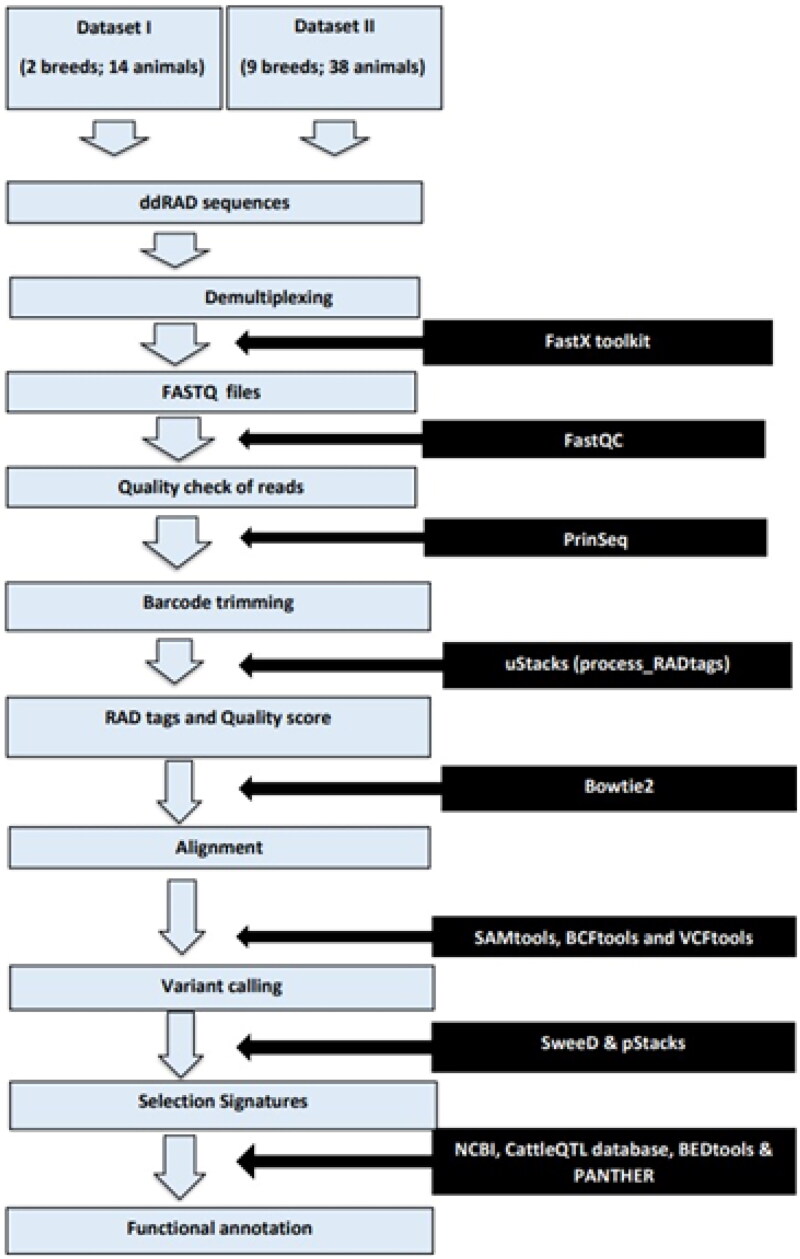
Workflow for identification and annotation of selective sweeps.

**Figure 2.**
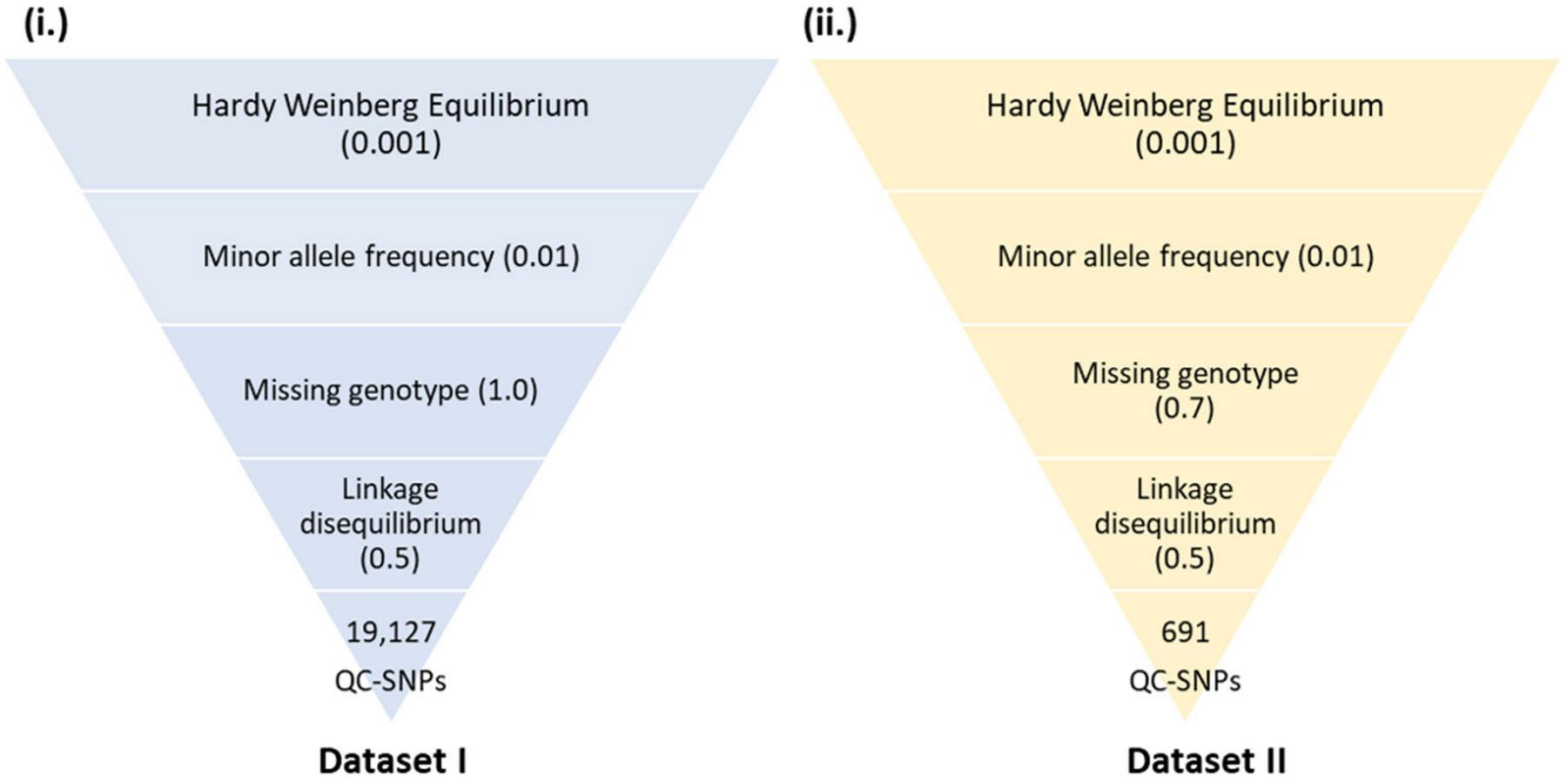
Quality control of SNPs in Dataset-I and Dataset-II.

General bioinformatics pipeline was used for data preprocessing (Fig. 1). Dataset-I (S1) consists of a total of 14 ddRAD sequences sampled from seven Gir and seven Tharparkar adult and unrelated cows. The sampled cows were affiliated with the breed standards laid by the National Bureau of Animal Genetic Resources (NBAGR), the nodal agency for breed registration in India. The above 14 samples were sequenced using the Illumina^®^ platform and deposited in NCBI (accessions: PRJNA678112 and PRJNA633222). The raw reads were retrieved and demultiplexed using FastX Toolkit v0.0.13 (http://hannonlab.cshl.edu/fastx_toolkit/).^35^ Preliminary screening of raw reads was done using FastQC v0.11.8.^36^ PRINSEQ-lite v0.20.4^37^ was used to skim off barcodes and adapter sequences trailing restriction sites. STACKS v2.2^38^ was used to process RAD tags and to remove reads having Phred score below 15. BOWTIE2 v2.3.4.1^39^ was used to map the query sequences to the latest reference assembly of *Bos taurus* (GCF_002263795.2^40^) and to *Bos indicus* (GCF_000247795.1 Bos indicus_1.0) assembly, in local very sensitive alignment mode. Gir reads were additionally mapped to reference assembly of Gir cattle (GCA_002933975.1) to enumerate additional variants in Gir cattle. SAM files were obtained, which were further processed to BAM files, using SAMtools v1.9.^41^ With the help of sort, index and mpileup in BCFtools v1.17^42^ and VCFtools v0.1.15,^42^ variants shared between the two breeds were inferred based on likelihood algorithms. Eventually, the total number of SNPs and InDels as compared to different reference genomes were filtered at read depth (RD) ≥ 10^43^ and mapping quality (MQ) ≥ 30^42^ using VCFtools v0.1.15. SNPs were subjected to quality checking (QC) for Hardy–Weinberg equilibrium (HWE; *p* < 0.001), minor allele frequency (MAF < 0.01), missing genotypes (MG < 1.0) and linkage disequilibrium (LD > 0.50; 50 site-window) using PLINK v1.07^44^ (Dataset-I; Fig. 2(a)).

### Detection of selection signatures

For intra-breed selection signatures, CLR values were obtained in Gir and Tharparkar, respectively using SweeD 3.0^45^ based on the hidden Markov model. SNPs were split breed-wise and chromosome-wise using BCFtools v1.16, and then SweeD 3.0 was run. Grid values were set as per the number of SNPs in each chromosome to get CLR at each SNP site, ranging from 393 to 980. Later, all the breed-wise CLR values were concatenated. For inter-breed selection signatures, population STACKS v2.61 yielded smoothed *F*_ST_ values between the two breeds. For both of the approaches, top 1 percentile SNPs (Dataset-I) with a window size of 10 kb, i.e., flanking the outlier SNPs ±5 kb both upstream and downstream, were assumed as putative selection signatures. Manhattan and circos plots were constructed using the qqman package in R^46^ and shinyCircos v2.0,^47^ respectively in R. The candidate genes under selection were traced in the coordinates of the windows of selection signatures.

To compare the reliability of the *F*_ST_ estimate, genotyping-by-sequencing (GBS) sequences of 24 unrelated adult cows (PRJNA400567) belonging to 8 breeds, *viz.,* Gangatiri, Hariana, Kankrej, Ongole, Sahiwal, Siri and Tharparkar breeds of zebu cattle as well as a Holstein cross, already generated by our collaborating partners^43^ were also included in the study. Thus, Dataset-II comprised 14 ddRAD sequences of Gir (zebu) (S1) and Tharparkar (zebu) (S1) as well as GBS sequences of 8 breeds (zebu and nonzebu) (S11). SNPs were called jointly from Dataset-II at RD ≥ 10 and MQ ≥ 30 subjected to QC for HWE (*p* < 0.001), MAF (0.01), LD (0.5) and MG (0.7). *F*_ST_ value was estimated again from the high-quality SNPs common to the nine breeds including Gir and Tharparkar (Dataset-II) using population STACKS v2.61 (Fig. 2(b)).

### Annotation of selection signatures

The general feature format files containing gene and quantitative trait loci (QTL) information were retrieved from the NCBI (https://www.ncbi.nlm.nih.gov/assembly/GCF_002263795.2) and CattleQTLdb,^48^ respectively. Candidate genes and QTL were annotated to BED files containing coordinates of selection signatures obtained from Dataset-I, using BEDtools v2.30.0.^49^ Graphical maps were generated using MG2C^50^ to address signatures on various chromosomes in relation to different traits. Genes common across CLR and *F*_ST_ approaches were traced using GeneVenn.^51^ The candidate genes were submitted as an input to the PANTHER Database for pathway analysis.^52^ Gene ontology was done by biological process (BP) terms, molecular functions terms as well as functional pathways.

## Results

### SNP identification

Mean base pair length of all the reads was 151 bp each. A total of 26 million raw reads were obtained from the Illumina platform comprising 13.67 million and 12.33 million of Gir and Tharparkar cattle respectively (S2). On an average, each sample produced 1.85 million reads. Gir samples had an average read count of 1.95 million while Tharparkar samples averaged a read count of 1.67 million. A total of 25.56 million (98.27%) clean reads passed the quality check (S2). When mapped to *B. taurus* assembly, the overall alignment rates were 99.85% and 99.87% in Gir and Tharparkar cattle, respectively. However, when mapped to *B. indicus* assembly, the overall alignment rates were 93.5% and 92.13% in Gir and Tharparkar cattle, respectively. Gir reads showed an alignment rate of 91.6% with Gir reference as compared to alignments with other assemblies; (S2). In all, 19,127 high-quality SNPs were obtained at RD ≥10 and MQ ≥30 (Fig. 2(a,b); S3), when mapped to *B. taurus* genome (Dataset-I) that were common across Gir and Tharparkar cattle to identify causal selection signatures. The Pearson’s correlation coefficient between length of autosomes and the number of SNPs was 0.93 (S4).

### Identification of selective sweeps

**Figure 3.**
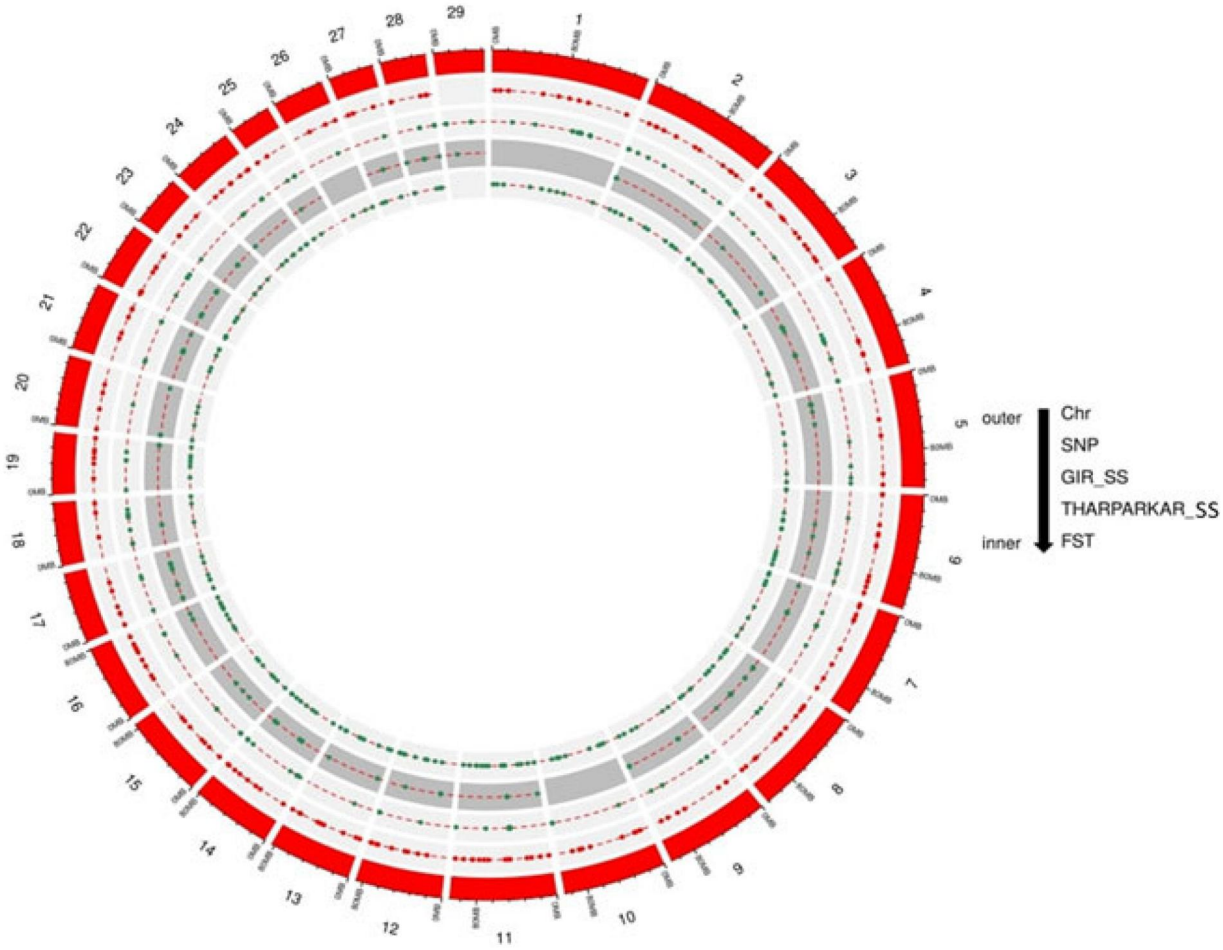
Circos plot showing SNPs and selective sweep identified in Gir and Tharparkar cattle. Outermost circle represents chromosomes, followed by number of SNPs identified, selection signatures in Gir using CLR statistic and selection signatures in Tharparkar using CLR statistic *F*_ST_.

**Figure 4.**
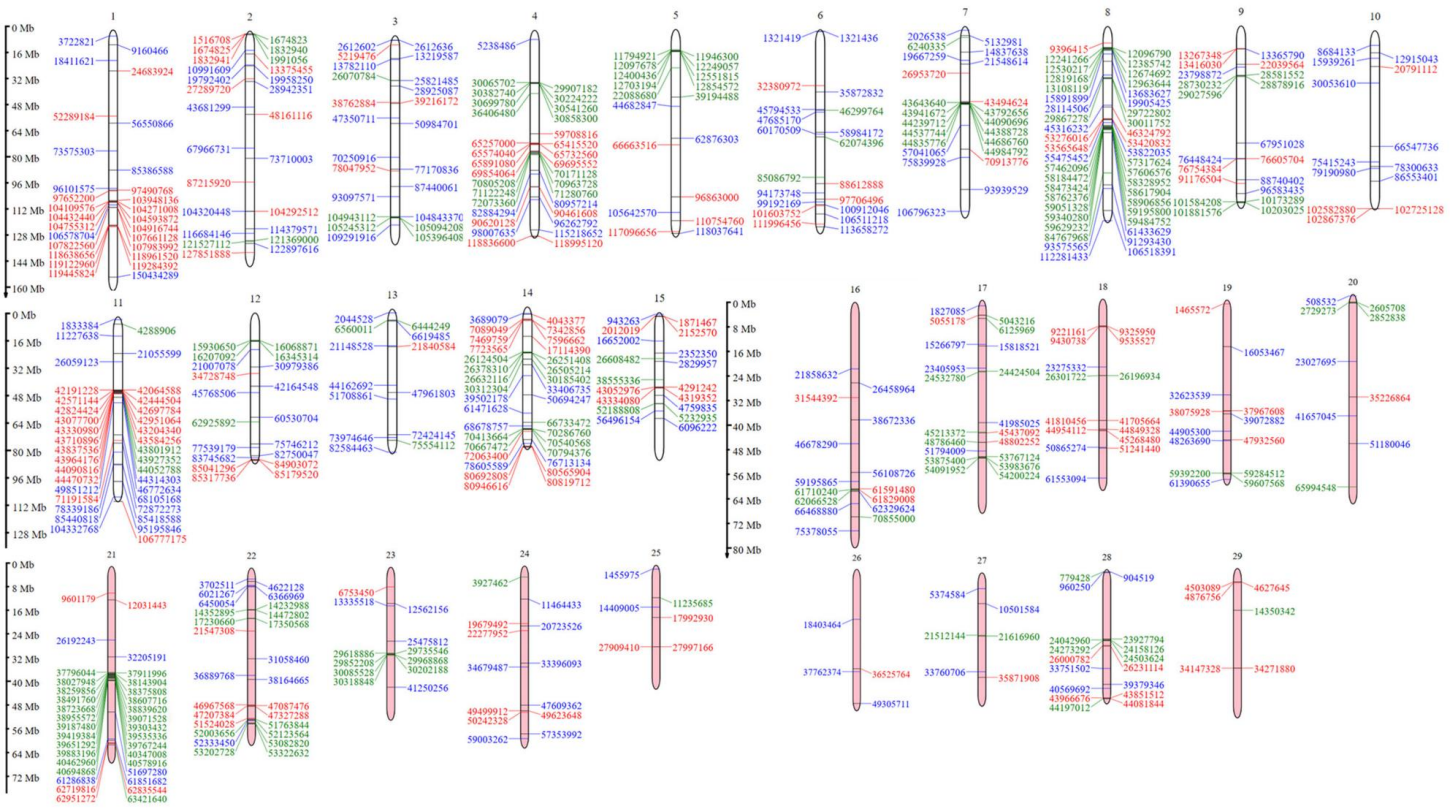
Chromosome map of selective sweep regions, where red denotes Gir cattle, green colour denotes Tharparkar cattle and blue colour denotes *F*_ST_.

**Figure 5.**
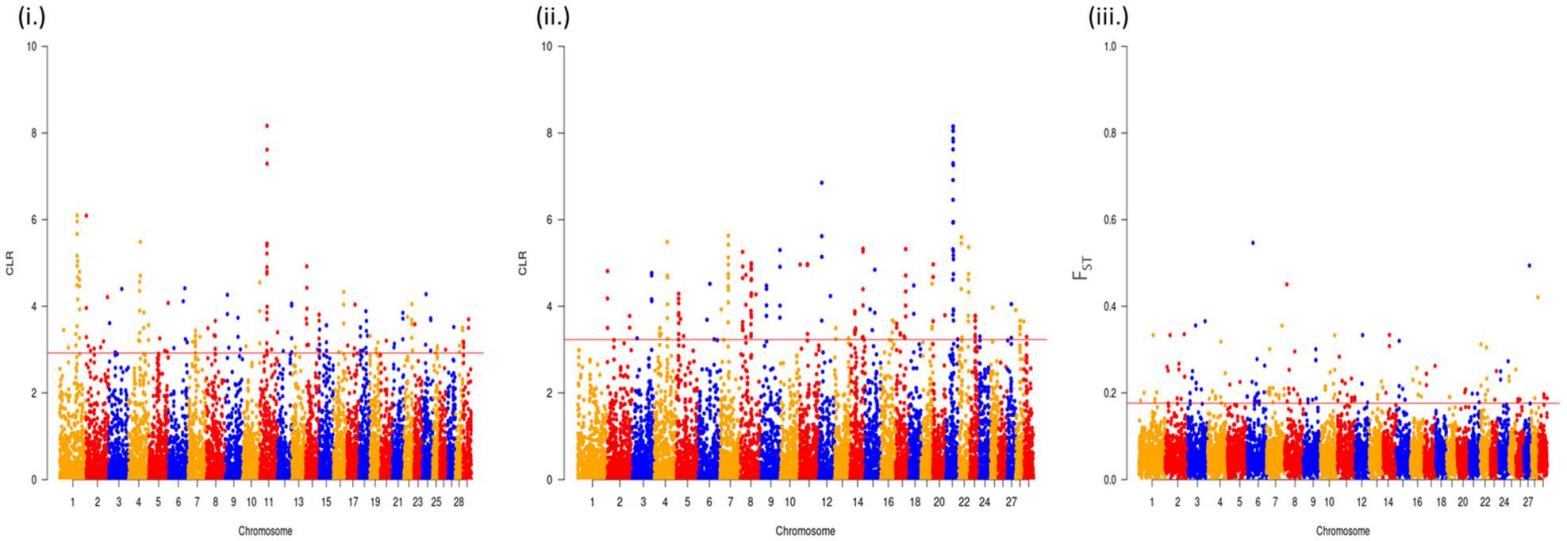
Manhattan plots to represent selective sweep regions detected by (i) CLR approach in Gir, (ii) CLR approach in Tharparkar and (iii) *F*_ST_ approach in Gir and Tharparkar.

A total of 191 sweep regions were found from Dataset-I by each of the approaches (Figs. 3 and 4). Based on CLR values, the top 1% variants crossing the threshold, *viz.,* CLR of 2.92 in Gir and 3.23 in Tharparkar cattle (Fig. 5) were considered as selection signatures. A total of 91 and 80 genes were traced in selective sweep regions with a window size of 10 kb (S12–S13) in Gir and Tharparkar cattle respectively. Signals of selection detected through CLR approach were found to overlap with *CACNA1D*, *CP*, *GNA14*, *LCP1*, *NFIB*, etc., genes having a role in production, *NOSTRIN*, *GHRHR*, *PRKD1* and *XCR1* genes having a role in reproduction functions. Likewise, *PARG*, *ROBO2*, *NOD1*, *IL12B*, etc., genes found to have a role in immunity (S8–S10). Gir and Tharparkar cattle shared seven genes found in selective sweep regions namely, *ARHGEF4*, *MUM1*, *FSTL4*, *GCFC2*, *TMEM132C*, *GINS3* and *MAP4* (S15).

The average *F*_ST_ value between the two populations was estimated to be 0.055. The top 1% variants with *F*_ST_ more than 0.66 are considered as the threshold for finding selection signatures. The outlier fraction contained 191 sites, flanked by a 10 kb  window, 5 kb up- and down- stream were declared as the putative selective sweep regions. A total of 100 genes were traced in the 191 sites (S14). Among these, selection signatures identified through *F*_ST_ approach overlapped with *ELOVL5*, *FAM13A*, *FHOD3*, *KCNK*, etc., genes were related to production traits, *MAPK10*, *RBMS3*, *ABCC1*, *TGFB2*, *ESR1*, *CACNA1D*, *CLEC 18 C*, *APAF1*, etc., genes have a role in reproduction. While genes like *RBS6KA2* and *FAM13A* have a role in immunity, the *RNF20* gene identified by the *F*_ST_ approach had a role in heat tolerance (S8–S10).

### Signatures associated with genes, QTL and pathways

**Figure 6.**
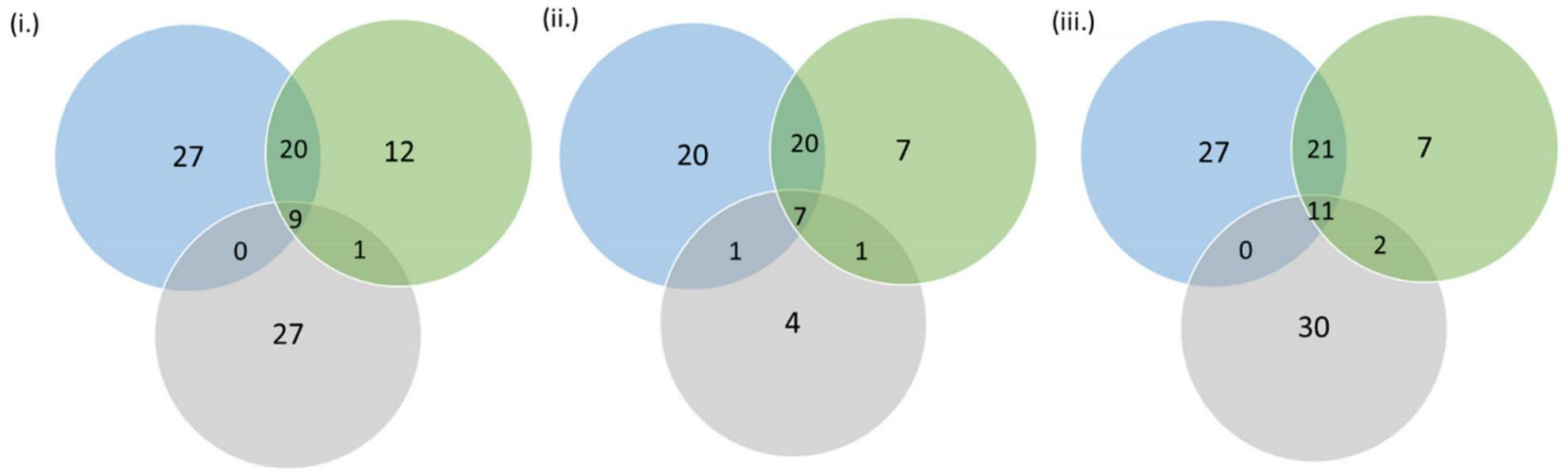
Venn diagram indicating genes overlapping for BPs, molecular function and pathways identified by (i) CLR in Gir, (ii) CLR in Tharparkar and (iii) *F*_ST_ in Gir and Tharparkar. Here, blue, green and grey colours depict BPs, molecular functions and gene pathways, respectively.

A total of 91, 80 and 100 genes were traced in selection signatures (S12–S14). After removing the common genes (S15), selection signatures overlapping 86 and 73 genes were detected as Gir- and Tharparkar- specific, respectively. A total of 32, 14 and 11 selection signatures were mapped to QTL associated with milk production, reproduction and growth traits respectively in Gir cattle (S16–S21). A total of 18 and 11 selection signatures were mapped to QTL related to milk production and reproduction traits respectively in Tharparkar cattle (S16–S21). Likewise, 38, 85 and 3 selection signatures were mapped to QTL related to milk production, reproduction and exterior traits respectively by *F*_ST_ approach (S22). Interestingly, the majority of the QTL responsible for milk casein percentage is found on the sixth chromosome (S16) and QTL responsible for reproduction traits on the X chromosome (S17). A total of 9, 7 and 11 genes overlapped with BPs, molecular function and pathways found by CLR approach in Gir, CLR approach in Tharparkar and *F*_ST_ approach in Gir-Tharparkar, respectively (Fig. 6; S23–S27). In all, 7 out of the 11 genes traced by *F*_ST_ approach were involved in apoptosis signalling pathway which is crucial for thermoregulation and adaptation. Greater number of genes were found to be enriched for Oxytocin receptor mediated signalling pathway (P04391), Wnt signalling pathway (P00057), Ras pathway (P04393), gonadotropin-releasing hormone receptor pathway (P06664) and insulin/IGF pathway-mitogen activated protein kinase/MAP kinase cascade (P00032) (S23–S27).

## Discussion

### SNP identification

Locally adapted breeds are the reservoirs of polymorphisms and signatures of selection *vis-a-vis* climate change.^26^ Breeds showing adaptive attributes are prioritized for conservation.^53^ Different studies have been published related to selection signatures in different Indian livestock species using SNP chip data, *viz.,* cattle,^54–57^ buffalo^57^ and sheep.^58^^,^^59^ Current study was undertaken to acknowledge selection hotspots in the genome of two zebu cattle, Gir and Tharparkar, which enriched them with excellent performance in hot and arid ecologies. Of note, cattle genetics and allied technologies such as SNP arrays and SNP databases developed so far show ascertainment bias^60–62^ and moreover, exclude discovery of breed-specific or rare variants.^63^^,^^64^ Nevertheless, there is always scope for finding some SNPs which may be static in exotic breeds while dynamic in indigenous breeds, at the same time. In the study strict quality control was followed, i.e., SNPs were called at RD ≥10 and MQ ≥30 subjected to HWE (0.001), MAF (0.01) and LD (0.5). However, there are growing concerns regarding the HWE test potentially excluding variants under selection, given its impact on signal detection.^65^ Recognizing that next-generation sequencing data can be prone to errors due to factors like base-calling and alignment, it is advisable to sequence target regions deeply (at >20× coverage) for accurate SNP calling. Nonetheless, to balance cost-effectiveness, sequencing in this study was performed at medium coverage (5–20×), which is commonly used. Consequently, to address issues stemming from low-quality scores, systematic disparities in quality scores for major and minor alleles, unusual LD patterns and extreme read depths, a filtering approach based on deviations from HWE was applied to enhance the precision of SNP calling,^66^ aligning with methodologies employed in other studies investigating positive selection in cattle using both whole genome resequencing^67^ and SNP chip data.^68^ GBS is more economical than SNP arrays for fewer individuals. Erstwhile works support similar sample size, *n* ≤ 7^32^^,^^43^^,^^45^^,^^69–71^ to ensure cost-effectiveness as well as animal welfare, while warranting accuracy and minimal false positives at the same time. STACKS was used for trimming low-quality reads as it checks the mean quality score using sliding windows while PRINSEQ trims across mean values.^72^ The reads were mapped to three different reference assemblies, to choose a reference genome yielding a higher number of polymorphisms for downstream analysis (S2). First, to infer indicine-specific variants,^32^ both the breeds under study were aligned with indicine reference assembly (Bos_indicus_1.0). This step minimizes ascertainment bias to taurine markers.^73^ Second, to have additional variants,^74^ Gir cattle were aligned with a Gir breed-specific reference assembly. However, due to the higher number of unmapped contigs present in this assembly,^75^ the alignment rates were found to be the lowest. In Tharparkar, breed-specific reference alignment was not carried out because no Tharparkar breed-specific reference assembly is available yet. Finally, SNPs identified with reference to ARS-UCD1.3,^40^ the latest, representative and more complete assembly, were used for downstream processing since it yielded higher variants (S3) than other assemblies. This was in line with Devadasan et al.,^32^ where Tharparkar reads were mapped to *B. indicus* (Bos_indicus_1.0) and *B. taurus* (Bos_taurus_UMD_3.1.1) reference genomes and ultimately, considered *B. taurus* alignment for downstream analysis as it yielded higher number of variants.

The 19,127 high-quality SNPs are lower than 87,047 SNPs reported in Tharparkar^32^ and 65,483 SNPs in Badri^33^ cattle, due to the application of stringent quality control. The number of SNPs are in line with Vineeth et al.^31^ and Jaglan et al.,^76^ where 193,803 and 18,056 SNPs were reported in 10 Sahiwal cattle and 96 Murrah buffaloes, respectively. However, SNPs reported are more than 9638 SNPs reported in 96 Vrindavani crossbred cattle^34^ implying greater degree of polymorphism in Gir. Gurgul et al.^77^ reported 8065 high-confidence SNPs in 48 cattle using the same technique. Malik et al.^43^ found 107,488 SNPs in 24 animals from eight cattle breeds, using GBS. The discrepancy in number is due to the use of the GBS approach which employs one single restriction enzyme as compared to dual frequent and rare cutters in ddRAD.^72^ The inter-SNP distance was higher than reported by Wang et al.^78^ More distanced SNPs reflect more stringent quality filtration. In the current study, the maximum and minimum number of SNPs was located at BTA1 and BTA28, respectively in both the breeds (S4). As per Wang et al.,^78^ the number of SNPs was most frequent in BTA11 while least frequent in BTA28. These differences in the distribution of SNPs may be due to differences in read depth threshold or may even be a breed-specific characteristic.^79^ Pearson’s coefficient of correlation was fairly high and concordant with the findings of Vineeth et al.^31^ Number of deletions was greater than the number of insertions.^80^ This may be due to the higher propensity of software to identify a missing segment as compared to the amplified segment.^81^ Since CLR and *F*_ST_ statistics are not haplotype-based, LD pruning was done to remove collinearity between SNPs^82^ using conservative thresholds (*r*^2^ > 0.5) over a sliding window of 50 bp.

### Detection of selection signatures

Detection of selection signatures by combinatorial approach is typically recommended.^83^ For that reason, CLR values and *F*_ST_ approach were used, similar to Wang et al.,^78^ to capture selection signatures within and between the breeds. Use of alternative approaches may complement and/or supplement our findings by finding sweeps which the former approach could not as well as by establishing congruence between the sweeps, making our findings reliable. However, as soon as selection comes into play, variability is evident in *F*_ST_ values. An outlier approach was used by sorting out the top 1 percentile of the polymorphic sites, to minimize false positives.^75^

**Figure 7.**
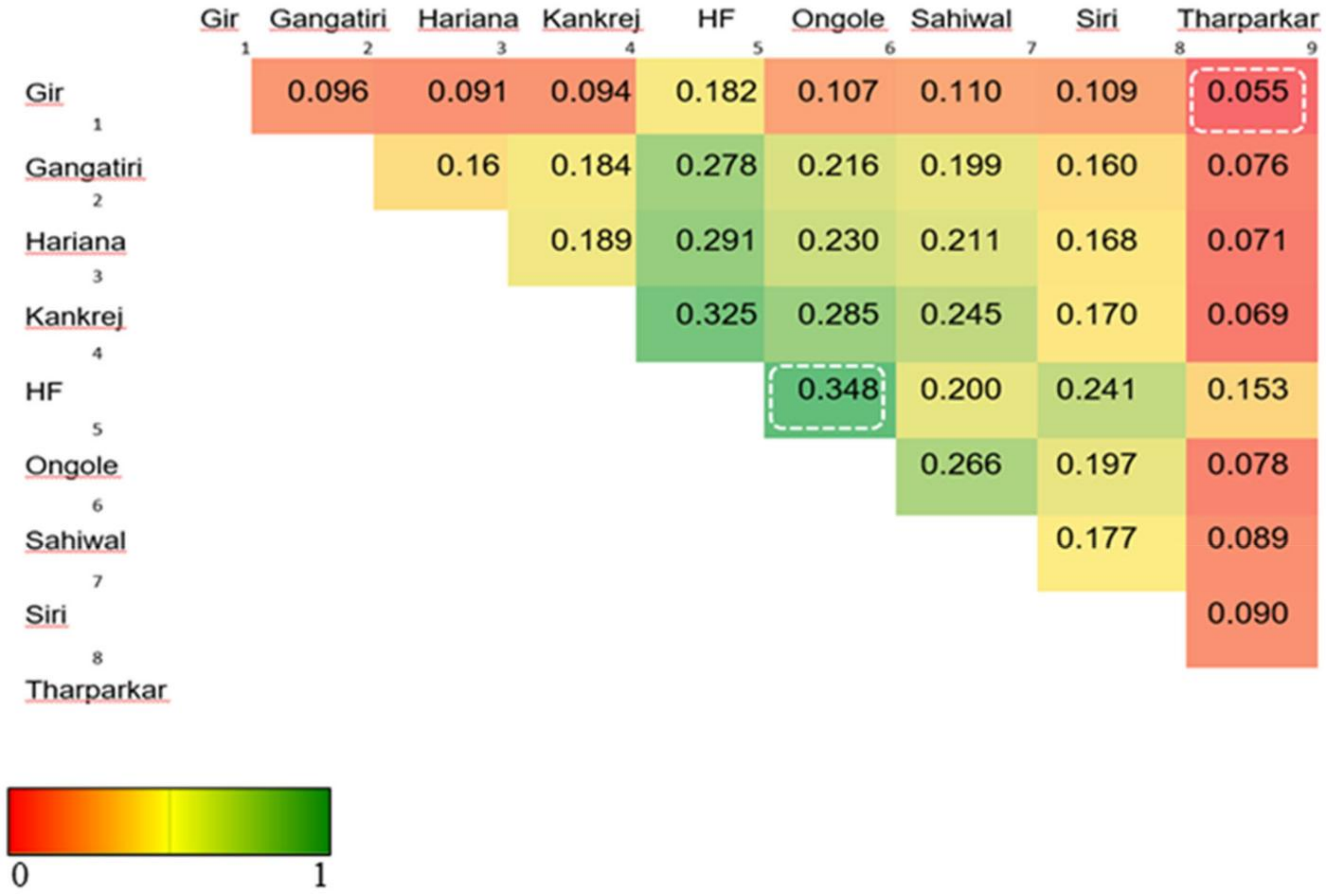
Heat map of *F*_ST_ values for pair-wise breed comparisons.

Lewontin and Krakauer^84^ suggested that under neutrality, only little heterogeneity in the form of *F*_ST_ is observed. *F*_ST_ based Population differentiation is a powerful approach to detect selection signatures for nonphenotyped populations.^85^ A systematic diversity investigation requires inclusion of more breeds for better reliability of estimates.^78^ Merging of datasets is not trivial, but differences in SNP identification codes and genomic coordinates can be harmonized across the datasets using the same reference genome assembly as well as strand orientation. Thus, to check the reliability of *F*_ST_, 24 cattle GBS sequences representing 8 breeds were merged with 14 ddRAD sequences representing Gir and Tharparkar breeds (S11). For merging, the datasets to be combined should share at least one breed for commonality; Tharparkar was common between 14 ddRAD and 7 GBS sequences in this case. Here, as the number of breeds was increased, the MG threshold for QC in Dataset-II was kept more lenient, i.e., 0.70 similar to De Donato et al.,^63^ and upon filtering for common SNPs, the number of SNPs were less than Dataset-I. These comparisons were made to cross-check *F*_ST_ values obtained in two breeds (S7) versus nine breeds (Fig. 7). The least *F*_ST_ (=0.055) was found between Gir and Tharparkar implying a moderate amount of variability, i.e., 5.5% shared between the two breeds, while the remaining 94.5% can be attributed to the within-breed variability (Fig. 7; S11). Average *F*_ST_ value between Gir and Tharparkar breeds was found to be minimum among all breed pairs, as well as found to be concordant, i.e., 0.055, both in Dataset-I and Dataset-II, which indicates these two breeds are genetically similar compared to the rest of the breeds in Dataset-II. The genetic similarity between the two breeds as compared to the other breeds may be due to overlapping arid ecological niches as well as milch utilities of the two breeds.^86^ Since *F*_ST_ measures allele frequency differences between populations, this may give clues for the selection of populations/breeds for crossbreeding to exploit heterosis. This is because crossbreeding involves the mating of animals chosen that have complementary traits,^87^^,^^88^ and alleles associated with the complementariness of traits display maximum *F*_ST_.^89^ In contrast, intra-population selection statistics such as CLR in our study explain adaptability to a given environment or production system may be given consideration in selective breeding.

In this study, 191 selection signatures and 91 and 80 overlapping genes have been identified in Gir and Tharparkar cattle, respectively using CLR approach while 100 genes were identified using the *F*_ST_ approach (S12–S14). Saravanan et al.^68^ identified 34 and 23 genes in regions under positive selection using bovine 50K SNP chip data in Gir and Tharparkar, respectively with the help of iHS statistics. Rajawat et al.^58^^,^^59^ using bovine 50K SNP chip data and CLR approach identified 22 genes in Gir and 23 genes in Tharparkar. In this study, most of the selection signatures were found on BTA1 and BTA8 by CLR approach in Gir and Tharparkar, respectively. However, maximum signatures were found on BTA3 using the *F*_ST_ approach. These findings suggest higher selection pressure acting on chromosomes 3, 8 and 25 as indicated in chromomap (Fig. 4). Gir cattle showed a higher number of candidate genes in sweep regions than Tharparkar. This can be attributed to higher selection pressure acting on coding regions in Gir cattle. Overlap in the signals between the CLR and *F*_ST_ approaches rules out the chances of demographic events from the selection model. Less number of overlaps may be due to different criteria employed in both approaches (S15). Thus, the two approaches are complementary, rather than supplementary. Genes were annotated using taurine genomic backgrounds due to more elaborate and precise annotation reports.^90^ However, a number of gene desert regions also form a major share of selection signatures due to incomplete genome annotation.^75^^,^^91^

### Signatures overlapping with production related genes

Selection signatures were annotated with candidate genes for production traits (S8). In Gir cattle, the gene *CACNA1D* (calcium voltage-gated channel subunit alpha1 D) is found in a positively selected region, which performs muscle contraction hormone or neurotransmitter release.^92^ The *GHRHR* (growth hormone releasing hormone receptor) gene encodes a receptor for growth hormone-releasing hormone.^93^ *MAP2K4* (mitogen-activated protein kinase kinase 4) is involved in prolactin signalling and GPCR pathway. Other important genes, *viz.*, *CP*, *RAB20*, *MAP4*, *ARHGEF4*, *GHRHR*, *TMEM132C*, *LDB2*, *GNA14* and *GHRHR* genes were also found. Maiorano et al.^94^ also identified *ARHGEF4* under selection by using the *F*_ST_ approach, carrying dairy importance.^95^ In Tharparkar cattle, the genes co-inheriting with regions under positive selection were; *STARD10* (StAR related lipid transfer domain containing 10) involved in lipid transport and regulation of bile acid secretion,^96^ *RPS6KA2* (ribosomal protein S6 kinase A2), implicated in controlling cell growth and differentiation^97^ and *XCR1* (X-C motif chemokine receptor 1) involved in calcium-dependent signal transduction.^98^ *MAP4*, *ARHGEF4*, *TMEM132C*, *RPS6KA2*, *VCP*, *RAB2A*, *NFIB* and *CHD7* genes were also found. Relatively higher number of genes related to production using CLR were found in Gir (nine), than in Tharparkar (six) (S8), which may explain higher milk production in Gir compared to Tharparkar.

Selective signatures identified using *F*_ST_ approach overlapped with *CAPN5* (Calpain 5) involved in signal transduction in a variety of cellular processes,^99^ *ELOVL5* (ELOVL fatty acid elongase 5) involved in long-chain fatty acids production^100^ and *MAPK10* (mitogen-activated protein kinase 10) involved in proliferation, differentiation, transcription regulation and development (GC04M085990). Apart from that, *FHOD3*, *FAM13A*, *OSBPL10*, *TGFB2*, *RBMS3*, *ABCC1* and *GRB10* genes were traced. No intersection was seen with some of the major genes such as *LEP* and *DGAT1* which suggests that genes found under selection may bear linkage and/or pleiotropy with the candidate genes for milk production and other performance traits. *MARCH4* gene which is involved in vesicular transport between membrane compartments^101^ was traced commonly in sweep regions identified by CLR and *F*_ST_ approach, suggesting the joint action of positive and divergent selection on *MARCH4* at the same time. This finding is in accordance with the findings of Zhao et al.^102^

### Signatures overlapping with reproduction related genes

In Tharparkar, *PRKD1* (protein kinase D1) gene was found which is involved in Golgi body membrane integrity and transport, cell migration, differentiation and adhesion.^103^ By *F*_ST_ approach, *RBMS3* (RNA binding motif single stranded interacting protein 3) has been implicated in diverse functions, such as DNA replication, gene transcription, cell cycle progression and apoptosis.^104^ *ESR1* (estrogen receptor 1) regulates growth, metabolism, sexual development, gestation and other reproductive functions.^105^ *CLEC18C*, *APAF1*, *ZGLP1*, *GNAQ*, *MAPK10* and *TGFB2* genes were also traced.

### Signatures overlapping with disease related genes

In Gir, *NOSTRIN* (nitric oxide synthase trafficking inducer) regulates neurotransmission, inflammatory response and vascular homeostasis,^106^ *CDH13* (cadherin 13) is involved in cell adhesion mechanism,^107^ *IL12B* (interleukin 12B) mediates long-term protection to an intracellular pathogen^108^ and *FYB1* is involved in platelet activation and controls the expression of interleukin-2.^109^ *MAP4*, *PARG*, *ROBO2*, *NOD1* and *PLB1* genes were found to overlap with the selection signatures (S9). In Tharparkar, overlaps were seen with *VTCN1* (V-set domain containing T cell activation inhibitor 1), which interacts with ligand bound to T cell receptors,^110^ *RPS6KA2* (ribosomal protein S6 kinase A2) implicated in controlling cell growth and differentiation^111^ and *LCP1* (lymphocyte cytosolic protein 1) implicated in tumorigenesis in solid tissues.^112^ *TLE1*, *MAP4*, *XCR1* and *TMEM154* were also found in sweep regions. Using *F*_ST_ approach, the genes found the sweep regions were *GNAQ* (G protein subunit alpha Q) required for platelet activation and regulates B-cell selection,^113^ *IL12RB1* (interleukin 12 receptor subunit beta 1) to mediate mycobacterial and salmonella infection pathways^114^ and *ATRN* (attractin) to regulate the chemotactic activity of chemokines.^115^ *TRIM33*, *TGFB2*, *GUCY1A2*, *TOX2* and *FAM13A* were the other genes found. Saravanan et al.^68^ reported *NCR3* in Gir and *DEFB7* in Tharparkar for immune response as genes under positive selection.

### Signatures overlapping with adaptation related genes

In Gir, *ASL* (argininosuccinate lyase) required in the liver to detoxify ammonia via the urea cycle^116^ was found. In Tharparkar, *ADAM22* (ADAM metallopeptidase domain 22), implicated in a variety of BPs involving cell interactions and neurogenesis,^117^ was found, and *TLE1, SP4 and TMTC2* were also detected. *DNAJC12* was identified by CLR approach in Tharparkar cattle. Members of the DNAJ family serve as cofactors for heat shock protein 70 (Hsp70) and are related to heat stress response.^118^ Using the CLR approach, a relatively higher number of genes were found in Tharparkar (12) compared to Gir (9) (S10), which explains the plasticity of Tharparkar to different climates. Through *F*_ST_ approach, *RNF20* (ring finger protein 20), a putative tumour suppressor,^119^ *PLXNA4* (plexin A4) involved in the positive regulation of axonogenesis^120^ and *NCAM1* (neural cell adhesion molecule 1) required in the development of the nervous system.^121^ *SNTG2*, *MPP5*, *DOCK9*, *ASTN2*, *MAPK10*, *DSCAML1*, *LINGO2*, *PHLDB2* and *DISP1* were also traced (S10).

### Signatures overlapping with QTL and pathway analysis

Selection signatures had overlapped maximum with reproduction and production associated with QTL (S16–S22). This is parallel with QTL mapped with selection signatures reported by Maiorano et al.^94^ However, Dixit et al.^122^ reported a maximum number of QTL overlapping with selection signatures in production and carcass traits in four Indian dairy breeds including Gir and Tharparkar using bovine HD 777k chip data. The *TOX* gene, related to immunity and daughter pregnancy rate was found under selection by *F*_ST_ approach in this study and was also reported by Maiorano et al.^94^ in QTL associated with milk. *KCNIP1* and *ROBO1* identified by CLR in Tharparkar and *F*_ST_ approach in the current study were reported to overlap with QTL related to production and composition. Rajawat et al.^58^^,^^59^ reported genes under positive selection in QTL using CLR approach, *viz.*, *CAHD1* for calving ease, *PPP1R14C* for body conformation and *PHF2* for milk fat percentage in Tharparkar and *CNTN5* for milk fat and protein quality, *KLHL20* for somatic cell count and *CADPS* for conception rate in Gir cattle.

Gene pathway analysis was done to show the biological traffic of genes in synchrony rather than the action of a single candidate gene.^123^ A total of seven genes traced by *F*_ST_ approach were involved in Apoptosis signalling pathway which is crucial for thermoregulation and adaptation (S23–S27). A greater representation of genes in oxytocin receptor mediated signalling pathway (P04391) indicates role of signatures in milk production,^124^ in Wnt signalling pathway (P00057) for ageing process^125^ and in Ras pathway (P04393) for cell proliferation and differentiation, thus having a direct role in immunity.^126^ Likewise, over representation of genes in gonadotropin-releasing hormone receptor pathway (P06664) indicates role of signatures in reproduction.^127^ Signatures in insulin/IGF pathway-mitogen activated protein kinase kinase/MAP kinase cascade (P00032) have a role in energy metabolism^128^ in the arid adaptive environment. An excess of genes in glutamatergic pathways and Parkinson’s disease pathways reflect their role in domestication and nervous system adaptation.^129^^,^^130^

## Conclusions

Research on selective sweeps offers valuable insights into biologically significant genetic variations that lead to genetic divergence. Genomic sub-sampling methods like ddRAD-seq offer a cost-effective solution to mitigate ascertainment bias, addressing limitations posed by generic SNP chips and expensive WGS. A total of 19,127 SNPs were used to examine selection signatures and found 191 potential regions through CLR and *F*_ST_ statistics. Analysing allele frequency differences through *F*_ST_ offers valuable insights for selecting populations in crossbreeding, capitalizing on trait complementarity, while, considering intra-population selection statistics like CLR ensures adaptability to specific environments and optimizing selective breeding strategies. The corresponding genes overlapping with selection signatures are particularly associated with crucial aspects of production (*CACNA1D*, *CP*, *GNA14*, etc.), reproduction (*NOSTRIN*, *GHRHR*, *PRKD1*, etc.) and adaptation (*ASL*, *ADAM22*, *DNAJC12*, etc.) underscoring the potential for comprehensive improvements in these key areas. Hence Gir and Tharparkar breeds can be considered as models for identifying selection signatures in hot and arid regions. These observations hold the potential to furnish valuable knowledge for future functional genomic investigations, QTL mapping, genome-wide association studies, genomic selection, the creation of breed-specific SNP panels, gene editing and the implementation of breeding strategies, and conservation initiatives. It is important to note that additional research with a larger sample size is necessary to validate our findings.

## Supplementary Material

Supplemental Material

## Data Availability

All data generated or analysed during this study are included in this manuscript.
