# Supplementary material for: Genome-wide selection signatures address trait specific candidate genes in cattle indigenous to arid regions of India
Source: Anim Biotechnol. 2023 Dec 13;35(1):2290521. doi: 10.1080/10495398.2023.2290521 (PMC12674412; doi:10.1080/10495398.2023.2290521)
Supplement: Supplemental Material [file LABT_A_2290521_SM1201.docx]

**S1** List of breeds included in the study (Dataset-I)

| **Breeds** | **NCBI**  **Acc.** | **Type** | **Utility** | **Native tract** | **Breeding tract** | **Sample size** | **Sampling location** | **Latitude** | **Longitude** | **AFC**  **(months)** | **CI (months)** | **LY (Kg)** | **MF (%)** |
| --- | --- | --- | --- | --- | --- | --- | --- | --- | --- | --- | --- | --- | --- |
| Gir | PRJNA678112 | Zebu | Milch | Gujarat | Gujarat | 7 | LRC, NDRI, Karnal | 29°41'8.49"N | 76°59'25.74"E | 39-59 | 13.4 | 2110 | 4.6 |
| Tharparkar | PRJNA633222 | Zebu | Milch | Rajasthan | Gujarat and Rajasthan | 7 | LRC, NDRI, Karnal | 29°41'8.49"N | 76°59'25.74"E | 36.71-52.5 | 14.18 | 1749 | 4.88 |
| 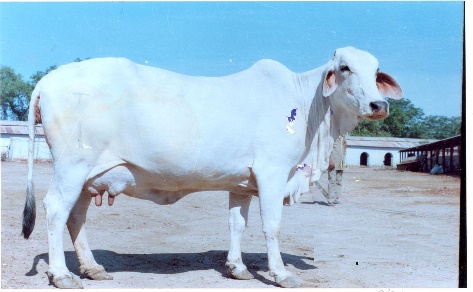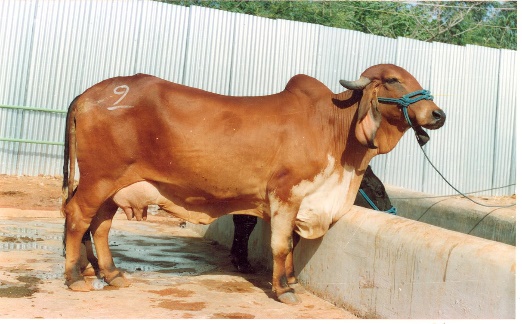 | | | | | | |  | | | | | | |
| **Gir female** | | | | | | | **Tharparkar female** | | | | | | |

**S2** Processing and alignment of reads (AR=Alignment rates, UAR=Unique Alignment rates)

|  | **Gir** | | | | | | | | | **Tharparkar** | | | | | | |
| --- | --- | --- | --- | --- | --- | --- | --- | --- | --- | --- | --- | --- | --- | --- | --- | --- |
|  |  | | | ***Bos taurus*** | | ***Bos indicus*** | | **Gir** | |  | | | ***Bos taurus*** | | ***Bos indicus*** | |
| **No.** | **ID** | **Raw reads** | **QC-passed reads** | **AR (%)** | **UAR (%)** | **AR (%)** | **UAR (%)** | **AR (%)** | **UAR (%)** | **ID** | **Raw reads** | **QC-passed reads** | **AR (%)** | **UAR (%)** | **AR (%)** | **UAR (%)** |
| 1 | G1 | 765970 | 754342 | 99.85 | 17.4 | 92.5 | 17.5 | 99.8 | 17.4 | T1 | 2507656 | 2465312 | 100 | 14.42 | 92.45 | 14.52 |
| 2 | G2 | 3310806 | 3223269 | 99.77 | 21.6 | 94.4 | 21.5 | 99.5 | 21.5 | T2 | 1889184 | 1858786 | 99.9 | 16.46 | 92.47 | 19.21 |
| 3 | G3 | 3305652 | 3249045 | 99.92 | 21.8 | 94.8 | 21.5 | 99.8 | 21.8 | T3 | 708670 | 698319 | 99.8 | 19.98 | 91.62 | 20.17 |
| 4 | G4 | 1283522 | 1264553 | 99.89 | 23 | 92.6 | 22.8 | 99.8 | 23 | T4 | 1721710 | 1696571 | 99.8 | 18.91 | 92.54 | 19.16 |
| 5 | G5 | 840808 | 829157 | 99.72 | 22.5 | 94.2 | 22.1 | 99.6 | 22.4 | T5 | 1617244 | 1592061 | 100 | 17.36 | 91.8 | 17.48 |
| 6 | G6 | 541836 | 533368 | 99.95 | 22.4 | 93.2 | 22 | 98.9 | 22.4 | T6 | 1406220 | 1381773 | 99.9 | 20.39 | 92.4 | 20.67 |
| 7 | G7 | 3626672 | 3572393 | 99.84 | 22.2 | 92.9 | 21.8 | 99.7 | 22.2 | T7 | 2481048 | 247734 | 99.9 | 18.84 | 92.4 | 19.25 |
| Total | | 13675266 | 13426127 | 99.85 | 21.6 | 93.5 | 21.6 | 91.6 | 21.5 | Total | 12331732 | 12140556 | 99.9 | 18.05 | 92.13 | 18.48 |

**S3** Distribution of variants at different read depths

|  | **Gir** | | | | | | | | | **Tharparkar** | | | | | |
| --- | --- | --- | --- | --- | --- | --- | --- | --- | --- | --- | --- | --- | --- | --- | --- |
|  | ***Bos taurus*** | | | ***Bos indicus*** | | | **Gir** | | | ***Bos taurus*** | | | ***Bos indicus*** | | |
| **RD** | **2** | **5** | **10** | **2** | **5** | **10** | **2** | **5** | **10** | **2** | **5** | **10** | **2** | **5** | **10** |
| **SNPs** | 198952 | 182917 | 163349 | 118245 | 109803 | 98747 | 54959 | 50747 | 45452 | 185682 | 167092 | 144417 | 57335 | 51246 | 43230 |
| **InDels** | 12603 | 11418 | 10074 | 11900 | 10774 | 9480 | 3384 | 3073 | 2735 | 13915 | 12496 | 10921 | 6652 | 5710 | 4632 |
| **Total** | 211555 | 194335 | 173423 | 130145 | 120577 | 108227 | 58343 | 53820 | 48187 | 199597 | 179588 | 155338 | 63987 | 56956 | 47682 |

**S4** Distribution of variants across different chromosomes

| **BTA** | **Length** | **SNPs** |
| --- | --- | --- |
| 1 | 158534110 | 980 |
| 2 | 136231102 | 861 |
| 3 | 121005158 | 791 |
| 4 | 120000601 | 752 |
| 5 | 120089316 | 788 |
| 6 | 117806340 | 634 |
| 7 | 110682743 | 735 |
| 8 | 113319770 | 778 |
| 9 | 105454467 | 697 |
| 10 | 103308737 | 726 |
| 11 | 106982474 | 841 |
| 12 | 87216183 | 619 |
| 13 | 83472345 | 714 |
| 14 | 82403003 | 645 |
| 15 | 85007780 | 605 |
| 16 | 81013979 | 681 |
| 17 | 73167244 | 664 |
| 18 | 65820629 | 623 |
| 19 | 63449741 | 586 |
| 20 | 71974595 | 579 |
| 21 | 69862954 | 596 |
| 22 | 60773035 | 495 |
| 23 | 52498615 | 444 |
| 24 | 62317253 | 498 |
| 25 | 42350435 | 474 |
| 26 | 51992305 | 419 |
| 27 | 45612108 | 429 |
| 28 | 45940150 | 393 |
| 29 | 51098607 | 407 |
| **Pearson’s correlation** | **0.93** | |

**S5** Selection signatures based on CLR in Gir

| **BTA** | **BP ±5 Kb** | **p-value** |
| --- | --- | --- |
| X | 105803176 | 1.93E-06 |
| X | 105332856 | 1.08E-06 |
| X | 81346528 | 1.07E-06 |
| X | 3273395 | 5.64E-07 |
| X | 3038235 | 3.71E-07 |
| X | 2803075 | 2.96E-07 |
| X | 2567915 | 2.56E-07 |
| X | 2332755 | 2.36E-07 |
| X | 2097595 | 2.24E-07 |
| X | 1862435 | 2.05E-07 |
| X | 1627275 | 1.93E-07 |
| X | 1392115 | 1.82E-07 |
| X | 1156955 | 1.67E-07 |
| X | 921795 | 1.60E-07 |
| X | 686635 | 1.49E-07 |
| X | 451475 | 1.40E-07 |
| 1 | 24683924 | 1.40E-05 |
| 1 | 118638656 | 3.39E-06 |
| 1 | 52289184 | 1.70E-06 |
| 1 | 118961520 | 1.28E-06 |
| 1 | 119122960 | 9.80E-07 |
| 1 | 97490768 | 9.56E-07 |
| 1 | 97652200 | 8.76E-07 |
| 1 | 119284392 | 8.51E-07 |
| 1 | 119445824 | 8.10E-07 |
| 1 | 104916744 | 7.32E-07 |
| 1 | 107983992 | 6.64E-07 |
| 1 | 107661128 | 6.19E-07 |
| 1 | 107822560 | 6.08E-07 |
| 1 | 104755312 | 5.64E-07 |
| 1 | 104593872 | 4.90E-07 |
| 1 | 104432440 | 4.35E-07 |
| 1 | 103948136 | 4.28E-07 |
| 1 | 104271008 | 4.17E-07 |
| 1 | 104109576 | 4.15E-07 |
| 2 | 104292512 | 1.30E-05 |
| 2 | 87215920 | 5.23E-06 |
| 2 | 127851888 | 4.93E-06 |
| 2 | 48161116 | 4.01E-06 |
| 2 | 1674825 | 1.90E-06 |
| 2 | 27289720 | 1.64E-06 |
| 2 | 1516708 | 1.63E-06 |
| 2 | 13375455 | 1.43E-06 |
| 2 | 1832941 | 1.40E-06 |
| 3 | 78047952 | 3.71E-06 |
| 3 | 38762884 | 2.23E-06 |
| 3 | 5219476 | 1.25E-06 |
| 3 | 39216172 | 9.80E-07 |
| 4 | 59708816 | 7.26E-06 |
| 4 | 90461608 | 2.40E-06 |
| 4 | 90620128 | 1.51E-06 |
| 4 | 65891080 | 1.38E-06 |
| 4 | 118836600 | 1.24E-06 |
| 4 | 118995120 | 1.04E-06 |
| 4 | 65732560 | 9.69E-07 |
| 4 | 65257000 | 9.46E-07 |
| 4 | 69854064 | 8.78E-07 |
| 4 | 65415520 | 8.68E-07 |
| 4 | 65574040 | 8.30E-07 |
| 4 | 69695552 | 8.13E-07 |
| 5 | 96863000 | 9.40E-06 |
| 5 | 117096656 | 1.55E-06 |
| 5 | 110754760 | 1.37E-06 |
| 5 | 66663516 | 1.29E-06 |
| 6 | 97706496 | 3.27E-06 |
| 6 | 101603752 | 2.80E-06 |
| 6 | 111996456 | 2.56E-06 |
| 6 | 32380972 | 2.43E-06 |
| 6 | 88612888 | 2.16E-06 |
| 7 | 70913776 | 8.91E-06 |
| 7 | 44984792 | 7.67E-06 |
| 7 | 44686760 | 3.27E-06 |
| 7 | 26953720 | 2.95E-06 |
| 7 | 44090696 | 5.02E-07 |
| 7 | 43941672 | 4.37E-07 |
| 7 | 43792656 | 4.19E-07 |
| 7 | 43494624 | 4.16E-07 |
| 7 | 43643640 | 4.11E-07 |
| 8 | 53565648 | 1.22E-05 |
| 8 | 9396415 | 2.23E-06 |
| 8 | 46324792 | 1.42E-06 |
| 8 | 53420832 | 1.19E-06 |
| 8 | 53276016 | 9.18E-07 |
| 9 | 13416030 | 2.18E-06 |
| 9 | 76754384 | 1.85E-06 |
| 9 | 13267348 | 1.83E-06 |
| 9 | 76605704 | 1.68E-06 |
| 9 | 22039564 | 1.58E-06 |
| 9 | 91176504 | 1.28E-06 |
| 10 | 20791112 | 5.37E-06 |
| 10 | 102725128 | 9.14E-07 |
| 10 | 102867376 | 8.98E-07 |
| 10 | 102582880 | 7.89E-07 |
| 11 | 71191584 | 2.75E-06 |
| 11 | 44090816 | 2.54E-06 |
| 11 | 44470732 | 1.15E-06 |
| 11 | 43964176 | 1.13E-06 |
| 11 | 106777175 | 8.95E-07 |
| 11 | 43837536 | 7.86E-07 |
| 11 | 43710896 | 6.19E-07 |
| 11 | 43584256 | 5.37E-07 |
| 11 | 43330980 | 4.26E-07 |
| 11 | 43204340 | 3.85E-07 |
| 11 | 43077700 | 3.45E-07 |
| 11 | 42951064 | 3.14E-07 |
| 11 | 42824424 | 3.02E-07 |
| 11 | 42697784 | 2.89E-07 |
| 11 | 42571144 | 2.83E-07 |
| 11 | 42444504 | 2.77E-07 |
| 11 | 42191228 | 2.66E-07 |
| 11 | 42064588 | 2.64E-07 |
| 12 | 84903072 | 2.56E-06 |
| 12 | 34728748 | 2.17E-06 |
| 12 | 85041296 | 1.76E-06 |
| 12 | 85317736 | 1.48E-06 |
| 12 | 85179520 | 1.39E-06 |
| 13 | 21840584 | 1.72E-06 |
| 14 | 7723565 | 3.61E-05 |
| 14 | 4043377 | 2.16E-05 |
| 14 | 17114390 | 4.69E-06 |
| 14 | 7089049 | 3.38E-06 |
| 14 | 72063400 | 2.67E-06 |
| 14 | 7596662 | 2.61E-06 |
| 14 | 7342856 | 2.32E-06 |
| 14 | 7469759 | 2.00E-06 |
| 14 | 80565904 | 8.65E-07 |
| 14 | 80946616 | 7.76E-07 |
| 14 | 80692808 | 7.57E-07 |
| 14 | 80819712 | 7.40E-07 |
| 15 | 2152570 | 1.94E-06 |
| 15 | 2012019 | 1.21E-06 |
| 15 | 1871467 | 9.94E-07 |
| 15 | 43334080 | 3.99E-07 |
| 15 | 42912424 | 3.81E-07 |
| 15 | 43193528 | 3.74E-07 |
| 15 | 43052976 | 3.74E-07 |
| 16 | 31544392 | 3.83E-06 |
| 16 | 61829008 | 1.52E-06 |
| 16 | 61710240 | 1.28E-06 |
| 16 | 61591480 | 1.15E-06 |
| 17 | 45437092 | 9.60E-06 |
| 17 | 48802252 | 5.51E-06 |
| 17 | 5055178 | 1.79E-06 |
| 18 | 51241440 | 1.10E-05 |
| 18 | 41705664 | 8.84E-06 |
| 18 | 26196934 | 5.56E-06 |
| 18 | 41810456 | 2.43E-06 |
| 18 | 44954112 | 1.92E-06 |
| 18 | 44849328 | 1.66E-06 |
| 18 | 9535527 | 9.77E-07 |
| 18 | 9221161 | 8.83E-07 |
| 18 | 9430738 | 8.61E-07 |
| 18 | 9325950 | 8.48E-07 |
| 19 | 37967608 | 2.54E-06 |
| 19 | 38075928 | 2.39E-06 |
| 19 | 47932560 | 1.23E-06 |
| 19 | 1465572 | 1.15E-06 |
| 20 | 35226864 | 6.16E-06 |
| 21 | 12031443 | 2.98E-05 |
| 21 | 9601179 | 1.10E-05 |
| 21 | 62719816 | 3.72E-06 |
| 21 | 62835544 | 2.16E-06 |
| 21 | 62951272 | 1.90E-06 |
| 22 | 51763844 | 6.40E-06 |
| 22 | 21547308 | 3.80E-06 |
| 22 | 51524028 | 3.76E-06 |
| 22 | 46967568 | 1.29E-06 |
| 22 | 47087476 | 1.16E-06 |
| 22 | 47207384 | 9.26E-07 |
| 22 | 47327288 | 8.39E-07 |
| 23 | 6753450 | 4.40E-06 |
| 24 | 22277952 | 3.70E-06 |
| 24 | 49623648 | 3.51E-06 |
| 24 | 49499912 | 2.08E-06 |
| 24 | 19679492 | 1.78E-06 |
| 25 | 50242328 | 2.64E-06 |
| 25 | 27997166 | 1.14E-06 |
| 25 | 27909410 | 1.11E-06 |
| 26 | 17992930 | 3.51E-06 |
| 26 | 36525764 | 2.30E-06 |
| 27 | 35871908 | 2.28E-06 |
| 28 | 26000782 | 4.21E-06 |
| 28 | 26231114 | 3.03E-06 |
| 28 | 44081844 | 1.77E-06 |
| 28 | 43966676 | 1.32E-06 |
| 28 | 43851512 | 1.20E-06 |
| 29 | 34271880 | 6.09E-06 |
| 29 | 34147328 | 3.91E-06 |
| 29 | 4876756 | 1.23E-06 |
| 29 | 4627645 | 1.01E-06 |
| 29 | 4503089 | 9.23E-07 |

**S6** Selection signatures based on CLR in Tharparkar

| **BTA** | **BP±5 Kb** | **p-value** |
| --- | --- | --- |
| 2 | 1674823 | 0.000107367 |
| 2 | 1832940 | 1.83718E-06 |
| 2 | 1991056 | 1.39863E-06 |
| 2 | 121527112 | 1.08867E-06 |
| 2 | 121369000 | 8.44268E-07 |
| 3 | 26070784 | 1.87938E-06 |
| 3 | 105396408 | 1.04246E-06 |
| 3 | 105245312 | 9.21203E-07 |
| 3 | 104943112 | 8.39545E-07 |
| 3 | 105094208 | 8.1502E-07 |
| 4 | 70171128 | 1.44013E-06 |
| 4 | 36406480 | 1.23908E-06 |
| 4 | 30858300 | 6.38107E-07 |
| 4 | 72073360 | 6.36151E-07 |
| 4 | 30699780 | 5.20732E-07 |
| 4 | 71280760 | 4.93927E-07 |
| 4 | 70805208 | 4.86208E-07 |
| 4 | 71122248 | 4.65754E-07 |
| 4 | 70963728 | 4.58091E-07 |
| 4 | 30541260 | 4.49786E-07 |
| 4 | 29907182 | 4.40611E-07 |
| 4 | 30382740 | 4.18956E-07 |
| 4 | 30065702 | 4.13331E-07 |
| 4 | 30224222 | 4.11121E-07 |
| 5 | 22088680 | 2.59757E-06 |
| 5 | 39194488 | 1.83037E-06 |
| 5 | 11946300 | 4.59775E-07 |
| 5 | 12097678 | 4.24049E-07 |
| 5 | 12249057 | 3.97468E-07 |
| 5 | 12400436 | 3.60188E-07 |
| 5 | 12551815 | 3.33375E-07 |
| 5 | 12703194 | 3.23055E-07 |
| 5 | 12854572 | 3.12322E-07 |
| 6 | 62074396 | 5.52055E-06 |
| 6 | 46299764 | 2.19745E-06 |
| 6 | 85086792 | 1.03485E-06 |
| 7 | 6240335 | 2.91803E-06 |
| 7 | 44984792 | 2.11558E-06 |
| 7 | 44835776 | 1.44435E-06 |
| 7 | 44686760 | 8.62825E-07 |
| 7 | 44537744 | 5.92876E-07 |
| 7 | 44388728 | 4.20277E-07 |
| 7 | 44239712 | 3.6586E-07 |
| 7 | 43643640 | 3.61222E-07 |
| 7 | 44090696 | 3.48598E-07 |
| 7 | 43792656 | 3.48545E-07 |
| 7 | 43941672 | 3.39332E-07 |
| 8 | 12096790 | 7.22663E-06 |
| 8 | 84767968 | 3.90226E-06 |
| 8 | 12241266 | 3.27173E-06 |
| 8 | 57606576 | 2.02795E-06 |
| 8 | 12385742 | 1.54339E-06 |
| 8 | 29867278 | 1.49547E-06 |
| 8 | 29722802 | 1.24099E-06 |
| 8 | 30011752 | 1.22203E-06 |
| 8 | 57462096 | 1.20008E-06 |
| 8 | 12530217 | 1.14074E-06 |
| 8 | 57317624 | 1.06348E-06 |
| 8 | 12674692 | 9.181E-07 |
| 8 | 12819168 | 8.0439E-07 |
| 8 | 12963644 | 7.12299E-07 |
| 8 | 13108119 | 6.61448E-07 |
| 8 | 58184472 | 5.86838E-07 |
| 8 | 58328952 | 4.76823E-07 |
| 8 | 58473424 | 4.14511E-07 |
| 8 | 59629232 | 3.87952E-07 |
| 8 | 58617904 | 3.7509E-07 |
| 8 | 59484752 | 3.62648E-07 |
| 8 | 58762376 | 3.57576E-07 |
| 8 | 59340280 | 3.539E-07 |
| 8 | 58906856 | 3.51746E-07 |
| 8 | 59195800 | 3.50545E-07 |
| 8 | 59051328 | 3.4443E-07 |
| 9 | 28581552 | 1.66204E-06 |
| 9 | 101584208 | 1.43391E-06 |
| 9 | 101732896 | 1.31018E-06 |
| 9 | 101881576 | 1.12587E-06 |
| 9 | 102030256 | 1.10732E-06 |
| 9 | 29027596 | 1.06057E-06 |
| 9 | 28730232 | 9.23448E-07 |
| 9 | 28878916 | 8.93281E-07 |
| 11 | 4288906 | 1.95135E-05 |
| 11 | 44052788 | 5.10564E-06 |
| 11 | 43927352 | 2.52148E-06 |
| 11 | 43801912 | 2.05074E-06 |
| 12 | 15930650 | 3.96656E-06 |
| 12 | 62925892 | 2.54741E-06 |
| 12 | 16068871 | 1.92234E-06 |
| 12 | 16207092 | 1.45587E-06 |
| 12 | 16345314 | 1.39298E-06 |
| 13 | 6560011 | 2.60192E-06 |
| 13 | 6444249 | 2.12852E-06 |
| 13 | 75554112 | 8.50465E-07 |
| 14 | 26632116 | 5.12159E-06 |
| 14 | 66733472 | 2.81601E-06 |
| 14 | 26505214 | 2.79533E-06 |
| 14 | 70794376 | 1.49447E-06 |
| 14 | 26378310 | 1.30012E-06 |
| 14 | 70667472 | 1.24898E-06 |
| 14 | 70540568 | 9.47688E-07 |
| 14 | 26251408 | 9.24312E-07 |
| 14 | 30312304 | 9.08888E-07 |
| 14 | 30185402 | 7.95849E-07 |
| 14 | 26124504 | 7.42818E-07 |
| 14 | 70413664 | 7.36885E-07 |
| 14 | 70286760 | 6.30508E-07 |
| 15 | 38555336 | 4.8879E-06 |
| 15 | 52188808 | 3.56973E-06 |
| 15 | 52329352 | 2.54431E-06 |
| 15 | 26608482 | 2.1197E-06 |
| 16 | 62066528 | 1.47388E-05 |
| 16 | 70855000 | 1.9074E-06 |
| 16 | 61710240 | 1.68636E-06 |
| 17 | 48786460 | 8.22493E-06 |
| 17 | 6125969 | 2.79219E-06 |
| 17 | 53983676 | 2.70716E-06 |
| 17 | 53875400 | 2.56481E-06 |
| 17 | 45213372 | 2.18455E-06 |
| 17 | 24424504 | 1.92819E-06 |
| 17 | 53767124 | 1.79988E-06 |
| 17 | 54091952 | 1.64926E-06 |
| 17 | 24532780 | 1.63527E-06 |
| 17 | 54200224 | 1.37075E-06 |
| 17 | 5043216 | 1.33721E-06 |
| 18 | 26196934 | 2.9622E-06 |
| 18 | 26301722 | 2.66807E-06 |
| 19 | 59607568 | 2.03964E-05 |
| 19 | 59392200 | 2.36983E-06 |
| 19 | 59284512 | 2.00267E-06 |
| 20 | 65994548 | 2.06922E-06 |
| 20 | 2605708 | 1.46621E-06 |
| 20 | 2852838 | 1.2713E-06 |
| 20 | 2729273 | 1.22222E-06 |
| 21 | 63421640 | 2.13037E-06 |
| 21 | 40694868 | 1.62115E-06 |
| 21 | 40578916 | 1.00468E-06 |
| 21 | 40462960 | 6.61592E-07 |
| 21 | 40347008 | 5.515E-07 |
| 21 | 39883196 | 3.42551E-07 |
| 21 | 39767244 | 3.28428E-07 |
| 21 | 39651292 | 3.17447E-07 |
| 21 | 37796044 | 3.11255E-07 |
| 21 | 39535336 | 2.93953E-07 |
| 21 | 37911996 | 2.87596E-07 |
| 21 | 39419384 | 2.83954E-07 |
| 21 | 38027948 | 2.75936E-07 |
| 21 | 39303432 | 2.7148E-07 |
| 21 | 38143904 | 2.65834E-07 |
| 21 | 39187480 | 2.61168E-07 |
| 21 | 38259856 | 2.57173E-07 |
| 21 | 39071528 | 2.55629E-07 |
| 21 | 38491760 | 2.54488E-07 |
| 21 | 38375808 | 2.52956E-07 |
| 21 | 38955572 | 2.528E-07 |
| 21 | 38839620 | 2.52572E-07 |
| 21 | 38607716 | 2.48672E-07 |
| 21 | 38723668 | 2.47917E-07 |
| 22 | 51763844 | 2.65773E-06 |
| 22 | 14472802 | 1.50248E-06 |
| 22 | 14352895 | 1.3377E-06 |
| 22 | 53322632 | 1.2832E-06 |
| 22 | 14232988 | 1.27893E-06 |
| 22 | 52123564 | 1.12577E-06 |
| 22 | 53202728 | 1.04538E-06 |
| 22 | 53082820 | 1.04231E-06 |
| 22 | 52003656 | 9.59361E-07 |
| 22 | 17350568 | 6.4246E-07 |
| 22 | 17230660 | 6.23264E-07 |
| 23 | 29618886 | 4.42289E-07 |
| 23 | 29735546 | 3.96158E-07 |
| 23 | 29852208 | 3.76455E-07 |
| 23 | 30318848 | 3.57574E-07 |
| 23 | 30085528 | 3.56763E-07 |
| 23 | 29968868 | 3.56647E-07 |
| 23 | 30202188 | 3.53291E-07 |
| 24 | 3927462 | 8.94745E-07 |
| 25 | 11235685 | 2.31084E-06 |
| 27 | 21616960 | 1.87452E-06 |
| 27 | 21512144 | 1.74789E-06 |
| 28 | 24273292 | 8.02707E-06 |
| 28 | 24158126 | 5.20993E-06 |
| 28 | 44197012 | 3.96651E-06 |
| 28 | 779428 | 3.83781E-06 |
| 28 | 24503624 | 2.09475E-06 |
| 28 | 24042960 | 2.05189E-06 |
| 28 | 23927794 | 1.41085E-06 |
| 29 | 14350342 | 8.08216E-07 |
| 30 | 132141056 | 7.41743E-06 |
| 30 | 10563317 | 7.38678E-07 |
| 30 | 10798477 | 6.35848E-07 |
| 30 | 11033637 | 6.04102E-07 |

**S7** Selection signatures based on F_ST_

| **BTA** | **BP±5 Kb** | **F_ST_** |
| --- | --- | --- |
| 1 | 73575303 | 0.47368 |
| 1 | 56550866 | 0.47368 |
| 1 | 1.5E+08 | 0.47368 |
| 1 | 3722821 | 0.43316 |
| 1 | 96101575 | 0.4 |
| 1 | 85386588 | 0.33333 |
| 1 | 1.07E+08 | 0.33333 |
| 1 | 9160466 | 0.33333 |
| 1 | 18411621 | 0.33333 |
| 2 | 1.04E+08 | 0.52083 |
| 2 | 1.23E+08 | 0.41538 |
| 2 | 1.14E+08 | 0.35556 |
| 2 | 67966731 | 0.35556 |
| 2 | 28942351 | 0.33333 |
| 2 | 10991609 | 0.33333 |
| 2 | 1.17E+08 | 0.33333 |
| 2 | 73710003 | 0.33333 |
| 2 | 19792402 | 0.33333 |
| 2 | 19958250 | 0.33333 |
| 3 | 25821485 | 0.52083 |
| 3 | 2612602 | 0.52083 |
| 3 | 1.05E+08 | 0.43316 |
| 3 | 50984701 | 0.43316 |
| 3 | 28925087 | 0.41538 |
| 3 | 47350711 | 0.35556 |
| 3 | 13782110 | 0.33333 |
| 3 | 87440061 | 0.33333 |
| 3 | 2612636 | 0.33333 |
| 3 | 13219587 | 0.33333 |
| 3 | 77170836 | 0.33333 |
| 3 | 1.09E+08 | 0.33333 |
| 3 | 70250916 | 0.33333 |
| 4 | 82884294 | 0.5102 |
| 4 | 1.15E+08 | 0.43316 |
| 4 | 98007635 | 0.4 |
| 4 | 96262792 | 0.4 |
| 4 | 80957214 | 0.33333 |
| 4 | 5238486 | 0.33333 |
| 5 | 44682847 | 0.43316 |
| 5 | 62876303 | 0.43316 |
| 5 | 1.18E+08 | 0.35556 |
| 5 | 1.06E+08 | 0.33333 |
| 6 | 35872832 | 0.55556 |
| 6 | 1321436 | 0.41538 |
| 6 | 47685170 | 0.41538 |
| 6 | 45794533 | 0.4 |
| 6 | 60170509 | 0.35556 |
| 6 | 1.07E+08 | 0.35556 |
| 6 | 58984172 | 0.35556 |
| 6 | 94173748 | 0.35556 |
| 6 | 1321419 | 0.33333 |
| 6 | 99192169 | 0.33333 |
| 6 | 1.01E+08 | 0.33333 |
| 7 | 93939529 | 0.55556 |
| 7 | 75839928 | 0.52083 |
| 7 | 57041065 | 0.4 |
| 7 | 2026538 | 0.35556 |
| 7 | 19667259 | 0.33333 |
| 7 | 21548614 | 0.33333 |
| 8 | 13683627 | 0.52083 |
| 8 | 55475452 | 0.4 |
| 8 | 15891899 | 0.4 |
| 8 | 1.12E+08 | 0.35556 |
| 8 | 61433629 | 0.33333 |
| 8 | 28114506 | 0.33333 |
| 8 | 93575565 | 0.33333 |
| 8 | 19905425 | 0.33333 |
| 8 | 53822035 | 0.33333 |
| 8 | 45316232 | 0.33333 |
| 8 | 1.07E+08 | 0.33333 |
| 9 | 76448424 | 0.5102 |
| 9 | 23798872 | 0.4 |
| 9 | 88740402 | 0.35556 |
| 10 | 13365790 | 0.35556 |
| 10 | 30053610 | 0.75 |
| 10 | 86553401 | 0.64706 |
| 10 | 79190980 | 0.41538 |
| 10 | 66547736 | 0.33333 |
| 10 | 75415243 | 0.33333 |
| 10 | 78300633 | 0.33333 |
| 10 | 15939261 | 0.33333 |
| 10 | 8684133 | 0.33333 |
| 11 | 44314303 | 0.64706 |
| 11 | 46772634 | 0.52083 |
| 11 | 11227638 | 0.5102 |
| 11 | 49851212 | 0.43316 |
| 11 | 72872273 | 0.41538 |
| 11 | 1833384 | 0.41538 |
| 11 | 85440818 | 0.35556 |
| 11 | 68105168 | 0.35556 |
| 11 | 85418588 | 0.33333 |
| 11 | 1.04E+08 | 0.33333 |
| 11 | 26059123 | 0.33333 |
| 11 | 95195846 | 0.33333 |
| 12 | 75746212 | 0.62051 |
| 12 | 77539179 | 0.43316 |
| 12 | 21007078 | 0.41538 |
| 12 | 42164548 | 0.4 |
| 12 | 83745682 | 0.35556 |
| 12 | 45768506 | 0.33333 |
| 12 | 60530704 | 0.33333 |
| 13 | 51708861 | 0.55556 |
| 13 | 73974646 | 0.52083 |
| 13 | 82584463 | 0.43316 |
| 13 | 44162692 | 0.4 |
| 13 | 72424145 | 0.4 |
| 13 | 6619485 | 0.35556 |
| 13 | 2044528 | 0.35556 |
| 13 | 21148528 | 0.33333 |
| 13 | 47961803 | 0.33333 |
| 14 | 39502178 | 0.64706 |
| 14 | 78605589 | 0.47368 |
| 14 | 33406735 | 0.43316 |
| 14 | 76713134 | 0.43316 |
| 14 | 68678757 | 0.4 |
| 14 | 61471628 | 0.4 |
| 14 | 3689079 | 0.4 |
| 14 | 50694247 | 0.33333 |
| 15 | 16652002 | 0.52083 |
| 15 | 60962223 | 0.41538 |
| 15 | 47598359 | 0.35556 |
| 15 | 28299579 | 0.35556 |
| 15 | 943263 | 0.33333 |
| 15 | 56496154 | 0.33333 |
| 15 | 23523500 | 0.33333 |
| 16 | 56108726 | 0.47368 |
| 16 | 46678290 | 0.41538 |
| 16 | 21858632 | 0.41538 |
| 16 | 75378055 | 0.4 |
| 16 | 66468880 | 0.35556 |
| 16 | 26458964 | 0.35556 |
| 16 | 62329624 | 0.35556 |
| 16 | 38672336 | 0.33333 |
| 17 | 15818521 | 0.43316 |
| 17 | 15266797 | 0.35556 |
| 17 | 41985025 | 0.35556 |
| 18 | 51794009 | 0.5102 |
| 18 | 1827085 | 0.41538 |
| 18 | 23405953 | 0.4 |
| 18 | 50865274 | 0.33333 |
| 18 | 23275332 | 0.33333 |
| 18 | 61553094 | 0.33333 |
| 19 | 16053467 | 0.35556 |
| 19 | 39072882 | 0.35556 |
| 19 | 32623539 | 0.35556 |
| 19 | 61390655 | 0.35556 |
| 19 | 44905300 | 0.33333 |
| 20 | 508532 | 0.47368 |
| 20 | 51180046 | 0.33333 |
| 20 | 23027695 | 0.33333 |
| 21 | 61851682 | 0.43316 |
| 21 | 32205191 | 0.4 |
| 21 | 51697280 | 0.33333 |
| 21 | 61286838 | 0.33333 |
| 21 | 26192243 | 0.33333 |
| 22 | 6366969 | 0.62051 |
| 22 | 31058460 | 0.47368 |
| 22 | 3702511 | 0.43316 |
| 22 | 4622128 | 0.43316 |
| 22 | 36889768 | 0.35556 |
| 22 | 38164665 | 0.35556 |
| 22 | 52333450 | 0.35556 |
| 22 | 6021267 | 0.35556 |
| 22 | 6450054 | 0.33333 |
| 23 | 41250256 | 0.55556 |
| 23 | 12562156 | 0.33333 |
| 23 | 13335518 | 0.33333 |
| 24 | 59003262 | 0.62051 |
| 24 | 11464433 | 0.52083 |
| 24 | 47609362 | 0.52083 |
| 24 | 33396093 | 0.43316 |
| 24 | 57353992 | 0.35556 |
| 24 | 34679487 | 0.35556 |
| 24 | 20723526 | 0.33333 |
| 25 | 1455975 | 0.35556 |
| 25 | 14409005 | 0.33333 |
| 26 | 37762374 | 0.41538 |
| 26 | 18403464 | 0.4 |
| 26 | 49305711 | 0.35556 |
| 27 | 33760706 | 0.55556 |
| 27 | 5374584 | 0.43316 |
| 27 | 10501584 | 0.33333 |
| 28 | 40569692 | 0.55556 |
| 28 | 904519 | 0.41538 |
| 28 | 39379346 | 0.35556 |
| 28 | 33751502 | 0.35556 |
| 28 | 960250 | 0.33333 |
| X | 1.37E+08 | 0.43316 |
| X | 81331743 | 0.35556 |
| X | 1.39E+08 | 0.35556 |
| X | 12840754 | 0.33333 |

**S8** Candidate genes related to production traits, which were found under selection (Here, g means Gir breed and t means Tharparkar breed)

| **Pubmed ID** | **Gene** | **Approach** | **Role** |
| --- | --- | --- | --- |
| 29454701 | *ELOVL5* | F_ST_ | Milk fat |
| 21257065 | *FAM13A* | F_ST_ | Milk yield and fat content |
| 26607727 | *FHOD3* | F_ST_ | Milk globulins |
| 26607727 | *KCNK* | F_ST_ | Milk globulins |
| 28318089 | *CACNA1D* | CLR (g) | Milk sugars |
| 19382692 | *CP* | CLR (g) | Milk glycoprotein |
| 28671246 | *GNA14* | CLR (g) | 305-day milk yield |
| 31360146 | *LCP1* | CLR (t) | Gluconeogenesis regulation |
| 30459810 | *NFIB* | CLR (t) | Milk production |
| 19554302 | *OSBPL10* | F_ST_ | Triglycerides |
| 31152389 | *RAB20* | CLR (g) | Mammary infection |
| 30611309 | *STARD10* | CLR (t) | Lipid transfer |
| 27148440 | *RPS6KA2* | CLR (t) | Mammary infection |
| 30185830 | *CDH13* | CLR (g) | Milk cholesterol |
| 29274975 | *FYB1* | CLR (g) | Udder index and milking speed |
| 28252361 | *MAP4* | CLR (g) | Milk production |
| 29495105 | *PLG1* | F_ST_ | Mammary development |
| 29447209 | *ARHGEF4* | CLR (t), CLR (g) | Milk yield |
| 30146276 | *GHRHR* | CLR (g) | Milk yield and quality |
| 21426999 | *XCR1* | CLR (t) | Production |

**S9** Candidate genes related to reproduction traits, which were found under selection (Here, g means Gir breed and t means Tharparkar breed)

| **Pubmed ID** | **Gene** | **Approach** | **Role** |
| --- | --- | --- | --- |
| 31849520 | *MAPK10* | F_ST_ | Cervical cancer |
| 28409548 | *RBMS3* | F_ST_ | Ovarian cancer |
| 29523764 | *ABCC1* | F_ST_ | Ovarian cancer |
| 24265800 | *TGFB2* | F_ST_ | Days open |
| 29807696 | *ESR1* | F_ST_ | Fertility |
| 26923315 | *CACNA1D* | F_ST_ | Daughter pregnancy rate |
| 30055605 | *CLEC18C* | F_ST_ | Fertility |
| 272897953 | *APAF1* | F_ST_ | Reproduction |
| 26020742 | *NOSTRIN* | CLR (g) | Uterine regulation |
| 31979402 | *GHRHR* | CLR (g) | Fertility |
| 26923368 | *PRKD1* | CLR (t) | Fertility |
| 21426999 | *XCR1* | CLR (t) | Fertility |
| 25510701 | *ANKRD57* | F_ST_ | Cumulus cells |
| 32054698 | *ZGLP1* | F_ST_ | Fertility |
| 22100599 | *TOX* | F_ST_ | Age at puberty |

**S10** Candidate genes related to Adaptation traits and Carcass traits, which were found under selection(Here, g means Gir and t means Tharparkar)

| **Pubmed ID** | **Gene** | **Approach** | **Role** |
| --- | --- | --- | --- |
| 20942903 | *GRB10* | F_ST_ | Growth |
| 22111664 | *PHLDB2* | F_ST_ | Neural control |
| 30608261 | *TLE1* | CLR (t) | Immunity |
| 29454820 | *IL12B* | CLR (g) | Mastitis |
| 28252361 | *MAP4* | CLR (t), CLR (g) | Mastitis |
| 21426999 | *XCR1* | CLR (t) | Mastitis |
| 27153114 | *CDH13* | CLR (g) | Immunity |
| 24403857 | *RBS6KA2* | CLR (t) | Immunity |
| 31164635 | *FAM13A* | F_ST_ | Lung functions |
| 29599514 | *IL12RB1* | F_ST_ | Immunity |
| 32029455 | *PARG* | CLR (g) | Immunity |
| 23663563 | *ROBO2* | CLR (g) | Immunity |
| 29778193 | *NOD1* | CLR (g) | Immunity |
| 19781038 | *IL12B* | CLR (g) | 19781038 |
| 31432180 | *MAPK10* | F_ST_ | Nervous System |
| 27127761 | *PLXNA4* | F_ST_ | Immunity |
| 23777392 | *TRIM33* | F_ST_ | Immunity |
| 20398313 | *TGFB2* | F_ST_ | Disease resistance |
| 31808517 | *DISP1* | F_ST_ | Nervous system |
| 25385143 | *VTCN1* | CLR (t) | Immunity |
| 23082221 | *TMEM154* | CLR (t) | Disease resistance |
| 26198991 | *ASL* | CLR (g) | Thermoregulation |
| 22567150 | *ATRN* | F_ST_ | UV protection |
| 25352127 | *TOX2* | F_ST_ | Immunity |
| 30423390 | *NCAM1* | F_ST_ | Nervous system |
| 31685652 | *DSCAML1* | F_ST_ | Nervous system |
| 29700853 | *LINGO2* | F_ST_ | Nervous System |
| 30649429 | *RNF20* | F_ST_ | Heat tolerance |
| 25410587 | *ASTN2* | F_ST_ | Nervous system |
| 27300296 | *GUCY1A2* | F_ST_ | Gastronematodal Burden |
| 29564645 | *SNTG2* | F_ST_ | Nervous system |
| 15558731 | *MPP5* | F_ST_ | Retinal function |
| 30816092 | *DOCK9* | F_ST_ | Nervous system |
| 2604820 | *SP4* | CLR (t) | Nervous system |
| 27717669 | *ADAM22* | CLR (t) | Nervous system |
| 30665415 | *TMTC2* | CLR (t) | Nervous System |
| 26634535 | *VCP* | CLR (t) | Carcass traits |
| 25330174 | *GNAQ* | F_ST_ | Carcass traits |
| 20662159 | *CAPN5* | F_ST_ | Carcass traits |
| 26960694 | *ELOVL5* | F_ST_ | Carcass traits |
| 30417949 | *TMEM132C* | CLR (t) | Carcass traits |
| 23300296 | *RPS6KA2* | CLR (t) | Carcass traits |
| 23637794 | *LDB2* | CLR (g) | Carcass traits |

**S11** List of breeds and bioinformatics for checking reliability of F_ST_ estimates (Dataset II)

| **Breed** | **NCBI Acc.** | **Location of sampling** | **n** | **Raw reads** | **Processed reads** | **Overall AR (%)** |
| --- | --- | --- | --- | --- | --- | --- |
| Gangatiri | PRJNA400567 | NBAGR (Karnal, India) | 3 | 16050390 | 14129557 | 99.4 |
| Holstein cross | PRJNA400567 | NBAGR (Karnal, India) | 3 | 20767314 | 15152522 | 99.49 |
| Hariana | PRJNA400567 | NBAGR (Karnal, India) | 3 | 14281978 | 8357856 | 99.45 |
| Kankrej | PRJNA400567 | PDC (Meerut, India) | 3 | 3795687 | 3735536 | 99.14 |
| Sahiwal | PRJNA400567 | PDC (Meerut, India) | 3 | 2733887 | 2692133 | 99.37 |
| Ongole | PRJNA400567 | PDC (Meerut, India) | 3 | 2619939 | 2582148 | 98.49 |
| Siri | PRJNA400567 | NBAGR (Karnal, India) | 3 | 20942393 | 20626846 | 99.35 |
| Tharparkar | PRJNA633222 | NBAGR (Karnal, India) | 10* | 37921316 | 37305319 | 99.68 |
| Gir | PRJNA678112 | NDRI (Karnal, India) | 7** | 13675266 | 13426127 | 99.7 |
|  |  |  |  |  |  |  |
| *10 (3 from public domain+ 7 generated in lab) | | | | | | |
| ** (all 7 generated in lab) | | | | | | |

**S12** Genes annotated to selection signatures in Gir cattle

| **BTA** | **Gene** | **Gene ID** | **Nomenclature** |
| --- | --- | --- | --- |
| 1 | *ROBO2* | 534842 | Roundabout guidance receptor 2 |
| 1 | *BBX* | 533187 | HMG-box containing |
| 1 | *SCHIP1* | 535716 | Schwannomin interacting protein 1 |
| 1 | *WWTR1* | 614786 | WW domain containing transcription regulator 1 |
| 1 | *CP* | 514194 | Ceruloplasmin |
| 1 | *HLTF* | 539633 | Helicase like transcription factor |
| 1 | *TRNAK-UUU* | 539633 | Helicase like transcription factor |
| 2 | *ARHGEF4* | 505852 | Rho guanine nucleotide exchange factor (GEF) 4 |
| 2 | *NOSTRIN* | 521834 | Nitric oxide synthase trafficking |
| 2 | *MARCH4* | 527624 | Membrane-associated ring finger (C3HC4) 4 |
| 4 | *GPR141* | 538837 | G protein coupled receptor |
| 4 | *GHRHR* | 281782 | Growth hormone releasing Hormone receptor |
| 4 | *MINDY4* | 615509 | MINDY lysine 48 deubiquitinase 4 |
| 4 | *GARS* | 408010 | Glycyl-trna synthetase |
| 4 | *NOD1* | 781426 | Nucleotide binding oligomerization domain containing 1 |
| 4 | *PTPRN2* | 616515 | Protein Tyrosine phosphatase N2 |
| 5 | *PAH* | 510583 | Phenylalanine hydroxylase |
| 5 | *TBC1D22A* | 618784 | TBC1 domain family member 22A |
| 5 | *RPL3* | 515194 | Ribosomal protein L3 |
| 6 | *GRID2* | 536367 | Glutamate ionotropic receptor delta type subunit 2 |
| 6 | *SEC31A* | 531964 | SEC31 homolog A (S. Cerevisiae) |
| 6 | *PTPN13* | 282333 | Protein tyrosine phosphatase |
| 6 | *LDB2* | 617976 | LIM domain binding 2 |
| 7 | *MARCH3* | 520348 | Membrane associated ring-CH-type finger 3 |
| 7 | *ARID3A* | 511283 | AT rich interactive domain 3A (BRIGHT-like) |
| 7 | *MUM1* | 513471 | Melanoma associated antigen (mutated) 1 |
| 7 | *FSTL4* | 540809 | Follistatin like 4 |
| 7 | *IL12B* | 281857 | Interleukin 12B |
| 8 | *MAMDC2* | 788176 | MAM domain containing 2 |
| 8 | *VPS13A* | 519494 | Vacuolar protein sorting 13 homolog A |
| 8 | *GNA14* | 281789 | G protein subunit alpha 14 |
| 9 | *CD109* | 534043 | Cluster of differentiation 109 |
| 9 | *IBTK* | 540885 | Inhibitor of Bruton tyrosine kinase |
| 9 | *ABRACL* | 505914 | ABRA C-terminal like |
| 9 | *ECT2L* | 100141063 | Epithelial cell transforming 2 like |
| 9 | *CNKSR3* | 506781 | CNKSR family member 3 |
| 10 | *RPS6KA5* | 504408 | Ribosomal protein S6 kinase A5 |
| 10 | *CTDSPL2* | 540232 | CTD small phosphatase like 2 |
| 11 | *BCL11A* | 538680 | B cell CLL/lymphoma 11A |
| 11 | *PUS10* | 614986 | Pseudouridylate synthase 10 |
| 11 | *KIAA1841* | 538151 | Uncharacterized protein |
| 11 | *GCFC2* | 521363 | GC-Rich DNA binding factor 2 |
| 11 | *EVA1A* | 528994 | EVA1-homolog A |
| 11 | *SH3RF3* | 615501 | SH3 domain containing ring finger 3 |
| 11 | *PLB1* | 506508 | Phospholipase B1 |
| 11 | *CACNA1B* | 282410 | Calcium voltage-gated channel subunit alpha1 B |
| 12 | *COL4A1* | 282191 | Collagen type IV alpha 1 chain |
| 12 | *COL4A2* | 508632 | Gene collagen type IV alpha 2 chain |
| 12 | *RAB20* | 615760 | Member RAS oncogene family |
| 14 | *CHMP4C* | 507572 | Charged multivesicular body protein 4C |
| 15 | *GRIA4* | 529904 | Glutamate ionotropic receptor AMPA type subunit 4 |
| 15 | *IPO7* | 513707 | Importin 7 |
| 15 | *TMEM41B* | 616372 | Transmembrane protein 41B |
| 16 | *SMYD3* | 616050 | SET and MYND domain containing 3 |
| 16 | *ACBD6* | 618245 | Acyl-coa binding domain containing 6 |
| 16 | *XPR1* | 536550 | Xenotropic and polytropic retrovirus receptor 1 |
| 17 | *TMEM132C* | 512602 | Transmembrane protein 132C |
| 18 | *CDH13* | 512302 | Cadherin 13 |
| 18 | *GINS3* | 538943 | GINS complex subunit 3 |
| 18 | *UBA2* | 507005 | Ubiquitin like modifier activating enzyme 2 |
| 18 | *GRIK5* | 537698 | Glutamate ionotropic receptor kainate type subunit 5 |
| 19 | *SKAP1* | 506769 | Src Kinase associated phosphoprotein 1 |
| 19 | *MAP3K3* | 508943 | Mitogen activated protein kinase kinase kinase 3 |
| 20 | *FYB1* | 535347 | FYN binding protein 1 |
| 22 | *ITPR1* | 317697 | 5-trisphosphate receptor type 1 |
| 22 | *CHDH* | 505218 | Choline dehydrogenase |
| 22 | *CACNA1D* | 408013 | Calcium voltage-gated channel subunit alpha1 D |
| 22 | *MAP4* | 281295 | Microtubule associated protein 4 |
| 24 | *CELF4* | 535177 | CUGBP Elav-like family member 4 |
| 24 | *DTNA* | 541153 | Dystrobrevin alpha |
| 24 | *MYO5B* | 514078 | Myosin VB |
| 24 | *MAPK4* | 529183 | Mitogen-activated protein kinase 4 |
| 25 | *GP2* | 531420 | Glycoprotein 2 |
| 25 | *GUSB* | 515687 | Glucuronidase beta |
| 25 | *CRCP* | 507533 | CGRP receptor component |
| 25 | *VKORC1L1* | 506318 | Vitamin k Epoxide reductase complex subunit 1-like |
| 25 | *ASL* | 512771 | Argininosuccinate lyase |
| 26 | *GFRA1* | 534801 | GDNF family receptor alpha 1 |
| 27 | *ZMAT4* | 616397 | Zinc finger matrin-type 4 |
| 28 | *COL13A1* | 613849 | Collagen type XIII alpha 1 chain |
| 28 | *OGDHL* | 531398 | Oxoglutarate dehydrogenase like |
| 28 | *PARG* | 281377 | Poly(ADP-ribose) glycohydrolase |
| 29 | *OPCML* | 281957 | Opioid binding protein/cell adhesion molecule like |
| 30 | *SLC6A14* | 511661 | Solute carrier family 6 member 14 |
| 30 | *WDR44* | 286825 | WD repeat domain 44 |
| 30 | *DOCK11* | 537297 | Dedicator of cytokinesis 11 |
| 30 | *SYTL5* | 536666 | Synaptotagmin like 5 |
| 30 | *LANCL3* | 519239 | Lanc like 3 |
| 30 | *MIR1277* | 102465116 | Microrna 1277 |
| 30 | *MIR2284W* | 100526411 | Microrna 2284W |
| 30 | *KLHL13* | 528138 | Kelch like family member 13 |

**S13** Genes annotated to selection signatures in Tharparkar cattle

| **BTA** | **Gene** | **Gene ID** | **Nomenclature** |
| --- | --- | --- | --- |
| 2 | *ARHGEF4* | 505852 | Rho guanine nucleotide exchange factor (GEF) 4 |
| 3 | *VTCN1* | 539919 | V-set domain-containing T-cell activation inhibitor 1 |
| 3 | *SCMH1* | 540926 | Scm polycomb group protein homolog 1 |
| 3 | *KCNQ4* | 528741 | Potassium voltage gated channel subfamily Q member 4 |
| 4 | *SP4* | 523019 | Sp4 transcription factor |
| 4 | *ADAM22* | 523645 | ADAM metallopeptidase domain 22 |
| 4 | *CDCA7L* | 514631 | cell division cycle associated 7 like |
| 4 | *SEMA3A* | 537777 | semaphorin 3A |
| 4 | *OSBPL3* | 537304 | oxysterol binding protein like 3 |
| 4 | *GSDME* | 616398 | gasdermin E |
| 4 | *MPP6* | 535171 | MAGUK p55 Subfamily member 6 |
| 5 | *METTL25* | 513885 | Methyltransferase like 25 |
| 5 | *TMTC2* | 100337258 | Transmembrane O-Mannosyltransferase |
| 6 | *STIM2* | 541299 | Stromal interaction molecule 2 |
| 6 | *UGT2A1* | 511743 | UDP glucuronosyltransferase family 2 member A1 complex locus |
| 7 | *NWD1* | 537999 | NACHT and WD repeat domain containing 1 |
| 7 | *MUM1* | 513471 | melanoma associated antigen (mutated) 1 |
| 7 | *AFF4* | 541306 | AF4/FMR2 family member 4 |
| 7 | *FSTL4* | 540809 | Follistatin like 4 |
| 7 | *CSNK1G2* | 506060 | casein kinase 1 gamma 2 |
| 7 | *SCAMP4* | 538819 | secretory carrier membrane protein 4 |
| 8 | *NFIB* | 538474 | nuclear factor I B |
| 8 | *TLE1* | 512888 | transducin-like enhancer of split 1 (E(sp1) homolog |
| 8 | *FANCG* | 615352 | Fanconi anemia complementation group G |
| 8 | *UNC13B* | 530692 | unc-13 homolog B |
| 8 | *VCP* | 507345 | valosin containing protein |
| 8 | *FAM120A* | 507997 | family with sequence similarity 120A |
| 8 | *PIGO* | 783989 | Phosphatidylionositol Glycan anchor biosynthesis class O |
| 9 | *PKIB* | 615798 | cAMP-dependent protein kinase inhibitor beta |
| 9 | *SERINC1* | 539993 | serine incorporator 1 |
| 9 | *RPS6KA2* | 517953 | ribosomal protein S6 kinase A2 |
| 9 | *UNC93A* | 613535 | unc-93 homolog A |
| 11 | *TSGA10* | 536433 | Testis specific 10 |
| 11 | *GCFC2* | 521363 | GC rich DNA binding factor 2 |
| 12 | *ZC3H13* | 540735 | Zinc finger CCCH-type containing 13 |
| 12 | *LCP1* | 540990 | lymphocyte cytosolic protein 1 |
| 12 | *LRRC63* | 100848618 | Leucine rich repeat containing 63 |
| 13 | *EYA2* | 615264 | EYA transcriptional coactivator and phosphatase 2 |
| 14 | *RAB2A* | 508373 | RAB2A |
| 14 | *CHD7* | 533175 | Chromodomain helicase DNA binding protein 7 |
| 14 | *PDE7A* | 100849042 | Phosphodiesterase 7A |
| 15 | *SPON1* | 282866 | spondin 1 |
| 15 | *PDE2A* | 281971 | phosphodiesterase 2A |
| 15 | *STARD10* | 514624 | StAR related lipid transfer domain containing 10 |
| 16 | *KIAA1614* | 785141 | Uncharacterized protein |
| 16 | *BATF3* | 535008 | basic leucine zipper ATF-like transcription factor 3 |
| 17 | *FAM160A1* | 528939 | Family with sequence similarity 160 Member A1 |
| 17 | *RNF34* | 506764 | ring finger protein 34 |
| 17 | *TMEM154* | 510523 | Transmembrane protein 154 |
| 17 | *TMEM132C* | 512602 | Transmembrane protein132C |
| 17 | *KDM2B* | 614487 | Lysine demethylase 2B |
| 17 | *CAMKK2* | 509084 | calcium/calmodulin dependent protein kinase kinase 2 |
| 17 | *IFT81* | 519602 | intraflagellar transport 81 |
| 17 | *P2RX7* | 286814 | purinergic receptor P2X 7 |
| 18 | *GINS3* | 538943 | GINS complex subunit 3 |
| 18 | *NDRG4* | 515033 | NDRG family member 4 |
| 18 | *CNOT1* | 533968 | CCR4-NOT transcription complex subunit 1 |
| 18 | *SETD6* | 539651 | SET domain containing 6 |
| 20 | *KCNIP1* | 541458 | potassium voltage-gated channel interacting protein 1 |
| 20 | *RANBP17* | 100337114 | RAN Binding protein 17 |
| 21 | *PRKD1* | 533270 | Protein kinase D1 |
| 22 | *FYCO1* | 100139246 | FYVE and coiled coil domain autophagy adaptor 1 |
| 22 | *ULK4* | 100336480 | Unc-51 like kinase 4 |
| 22 | *TRAK1* | 531649 | trafficking protein |
| 22 | *THUMPD3* | 508940 | THUMP domain containing 3 |
| 22 | *SRGAP3* | 512892 | SLIT-ROBO Rho GTPase activating protein 3 |
| 22 | *MAP4* | 281295 | microtubule associated protein 4 |
| 22 | *SMARCC1* | 522045 | SWI/SNF related, matrix associated, Actin dependent regulator of chromatin subfamily C Member 1 |
| 22 | *XCR1* | 617880 | X-C motif chemokine receptor 1 |
| 23 | *TRNAA-AGC_12* | 112444062 | tRNA |
| 23 | *OR2J2* | 539623 | Olfactory receptor family 2 subfamily J Member 2 |
| 23 | *TRNAK-UUU_35* | 112444034 | tRNA |
| 24 | *ZNF407* | 530360 | Zinc finger protein 407 |
| 25 | *SNX29* | 518366 | sorting nexin 29 |
| 27 | *TUSC3* | 615007 | tumor suppressor candidate 3 |
| 28 | *CTNNA3* | 780777 | catenin alpha 3 |
| 28 | *DNAJC12* | 281259 | DnaJ heat shock protein family (Hsp40) member C12 |
| 28 | *HERC4* | 533336 | HECT and RLD domain containing E3 ubiquitin protein ligase 4 |
| 28 | *ZFAND4* | 514391 | Zinc finger AN1-type containing 4 |

**S14** Genes annotated to selective sweeps using F_ST_ Value

| **BTA** | **Genes** | **Gene ID** | **Gene name** |
| --- | --- | --- | --- |
| 1 | *PHLDB2* | 509784 | Pleckstrin homology like domain family B member 2 |
| 1 | *TNIK* | 539627 | TRAF2 and NCK interacting Kinase |
| 1 | *PPM1L* | 541235 | Protein phosphatase, Mg2+/Mn2+ dependent 1L |
| 2 | *FMNL2* | 788312 | Formin like 2 |
| 2 | *B3GALT1* | 539027 | Beta-1,3-galactosyltransferase 1 |
| 2 | *MARCH4* | 527624 | Membrane-associated ring finger (C3HC4) 4, E3 ubiquitin protein |
| 3 | *MAN1A2* | 532645 | Mannosidase alpha class 1A member 2 |
| 3 | *TRIM33* | 533296 | Tripartite motif containing 33 |
| 3 | *TNNI3K* | 535940 | TNNI3 interacting kinase |
| 3 | *WLS* | 528123 | Wntless Wnt ligand secretion mediator |
| 4 | *GRB10* | 407210 | Growth factor receptor bound protein 10 |
| 4 | *CDK13* | 511147 | Cyclin dependent kinase 13 |
| 4 | *PLXNA4* | 407104 | Plexin A4 |
| 5 | *APAF1* | 537782 | Apoptotic peptidase activating factor 1 |
| 6 | *FAM13A* | 282605 | Family with sequence similarity 13 member A |
| 6 | *RBPJ* | 767928 | Recombination signal binding protein for immunoglobulin kappa J |
| 6 | *PDS5A* | 533206 | PDS5 cohesin associated factor A |
| 6 | *MAPK10* | 537631 | Mitogen-activated protein kinase 10 |
| 6 | *HS3ST1* | 538691 | Heparan sulfate-glucosamine 3-sulfotransferase 1 |
| 6 | *SORCS2* | 618257 | Sortilin related Vps 10p domain containing receptor 2 |
| 7 | *ADAMTS2* | 282401 | ADAM metallopeptidase with thrombospondin type 1 motif 2 |
| 7 | *ICAM5* | 505158 | Intercellular adhesion molecule 5 |
| 7 | *FDX2* | 505159 | Ferredoxin2 |
| 7 | *IL12RB1* | 524299 | Interleukin 12 receptor subunit beta 1 |
| 7 | *ZGLP1* | 782656 | Zinc finger GATA like protein 1 |
| 7 | *UBXN6* | 507936 | UBX domain protein 6 |
| 7 | *KIAA0825* | 617013 | Uncharacterized protein |
| 8 | *LINGO2* | 539968 | Leucine rich repeat and Ig domain containing 2 |
| 8 | *TJP2* | 407101 | Tight junction protein 2 |
| 8 | *GNAQ* | 536654 | Guanine nucleotide binding protein (G protein), q polypeptide |
| 8 | *ZCCHC7* | 511821 | Zinc finger CCHC-type containing 7 |
| 8 | *RNF20* | 513326 | Ring finger protein 20 |
| 8 | *ASTN2* | 781555 | Astrotactin2 |
| 8 | *SNTG2* | 615686 | Syntrophin gamma 2 |
| 9 | *SOGA3* | 535392 | SOGA family memeber 3 |
| 9 | *ESR1* | 407238 | Estrogen receptor 1 |
| 9 | *PLG* | 280897 | Plasminogen |
| 10 | *MEGF11* | 100336680 | Protein_coding |
| 10 | *SCG5* | 508224 | Secretogranin V |
| 10 | *MPP5* | 528109 | Membrane palmitoylated protein 5 |
| 11 | *SMYD5* | 509313 | SMYD family member 5 |
| 11 | *GALM* | 616676 | Galactose mutarotase |
| 11 | *ABCG5* | 515536 | ATP-binding cassette, sub-family G (WHITE), member 5 |
| 11 | *SEP10* | 514603 | Septin 10 |
| 11 | *IL1F10* | 615702 | Interleukin 1 family member 10 |
| 11 | *TCF7L1* | 515303 | Transcription factor 7 like 1 |
| 11 | *KCNK3* | 519188 | Potassium two pore domain channel subfamily K member 3 |
| 11 | *ADAMTS13* | 532272 | ADAM metallopeptidase with thrombospondin type 1 motif 13 |
| 11 | *NOTO* | 100335771 | Notochord homeobox |
| 12 | *WDFY2* | 523432 | WD repeat and FYVE domain containing 2 |
| 12 | *SLC7A1* | 539465 | Solute carrier family 7-member 1 |
| 12 | *DOCK9* | 616045 | Dedicator of cytokinesis 9 |
| 13 | *PLCB4* | 281985 | Phospholipase C, beta 4 |
| 13 | *SPTLC3* | 100336940 | Serine palmitoyltransferase long chain base subunit 3 |
| 13 | *MALRD1* | 783682 | MAM and LDL receptor class A domain containing 1 |
| 13 | *SHLD1* | 615129 | Chromosome 13 c20orf196 homolog |
| 13 | *ATRN* | 281017 | Attractin |
| 13 | *TOX2* | 519845 | TOX high mobility group box family member 2 |
| 14 | *KCNK9* | 539586 | Potassium two pore domain channel subfamily K member 9 |
| 14 | *SULF1* | 535166 | Sulfatase1 |
| 15 | *GUCY1A2* | 535166 | Guanylate cyclase 1 soluble subunit alpha 2 |
| 15 | *NCAM1* | 281941 | Neural cell adhesion molecule 1 |
| 15 | *DSCAML1* | 538739 | DS cell adhesion molecule like 1 |
| 15 | *CAPN5* | 536988 | Calpain 5 |
| 16 | *TGFB2* | 534069 | Transforming growth factor beta 2 |
| 16 | *DISP1* | 534637 | Dispatched RND transporter family member 1 |
| 16 | *FMO3* | 281167 | Flavin containing monooxygenase 3 |
| 16 | *RABGAP1L* | 540101 | RAB gtpase activating protein 1 like |
| 17 | *INPP4B* | 534793 | Inositol polyphosphate-4-phosphatase type II B |
| 18 | *CLEC18C* | 516050 | C-type lectin domain family 18 member C |
| 19 | *ASIC2* | 617930 | Acid sensing ion channel subunit 2 |
| 19 | *ARHGAP23* | 523030 | Rho gtpase activating protein 23 |
| 19 | *ABCA10* | 504909 | ATP binding cassette subfamily A member 10 |
| 19 | *MAP3K14* | 508367 | Mitogen-activated protein kinase kinase kinase 14 |
| 19 | *ERN1* | 112442685 | Endoribonuclease IRE1 |
| 20 | *SLIT3* | 615883 | Slit guidance ligand 3 |
| 20 | *ANKRD55* | 533461 | Ankyrin repeat domain 55 |
| 20 | *PDZD2* | 516852 | PDZ domain containing 2 |
| 21 | *CTXND1* | 107131603 | Cortexin domain containing 1 |
| 21 | *TRNAG-CCC_135* | 112443404 | trna |
| 21 | *TSPAN3* | 616881 | Tetraspanin 3 |
| 21 | *AK7* | 511269 | Adenylate kinase 7 |
| 22 | *RBMS3* | 784766 | RNA binding motif single stranded interacting protein 3 |
| 22 | *TRNAG-CCC_137* | 112443568 | trna |
| 22 | *OSBPL10* | 507708 | Oxysterol binding protein like 10 |
| 22 | *ADAMTS9* | 537051 | ADAM metallopeptidase with thrombospondin type 1 motif 9 |
| 22 | *KLHL18* | 517831 | Kelch like family member 18 |
| 23 | *DNAH8* | 525414 | Dnah8 |
| 23 | *KIF6* | 104968668 | Kinesin family member 6 |
| 23 | *ELOVL5* | 617293 | ELOVL fatty acid elongase 5 |
| 24 | *FHOD3* | 785433 | Formin homology 2 domain containing 3 |
| 24 | *CABLES1* | 100138286 | Cdk5 and Abl enzyme substrate 1 |
| 24 | *GREB1L* | 535053 | GREB1 like retinoic acid receptor coactivator |
| 24 | *NEDD4L* | 510003 | NEDD4 like E3 ubiquitone protein ligase |
| 26 | *ABCC1* | 281588 | ATP binding cassette subfamily C member 1 |
| 26 | *SLIT1* | 510761 | Slit guidance ligand 1 |
| 28 | *CCSER2* | 540046 | Coiled-coil serine rich protein 2 |
| 28 | *GRID1* | 100337106 | Glutamate lonotropic receptor delta type subunit 1 |
| X | *GPC3* | 615239 | Glypican 3 |
| X | *DHRSX* | 513482 | Dehydrogenase/reductase x-linked |

**S15** Common genes under selection in Gir and Tharparkar breeds (F_ST_ approach and CLR approach)

| **BTA** | **Gene** | **GeneID** | **Gene** | **Role** |
| --- | --- | --- | --- | --- |
| 2 | *ARHGEF4* | 505852 | Rho guanine nucleotide exchange factor (GEF) 4 | Milk yield |
| 7 | *MUM1* | 513471 | melanoma associated antigen (mutated) 1 | Disease associated |
| 7 | *FSTL4* | 540809 | Follistatin Like 4 | Reproduction |
| 11 | *GCFC2* | 521363 | GCFC2 | Nervous system |
| 17 | *TMEM132C* | 512602 | TMEM132C | Carcass traits |
| 18 | *GINS3* | 538943 | GINS complex subunit 3 | DNA replication |
| 22 | *MAP4* | 281295 | microtubule associated protein 4 | Mastitis |

**S16** Selection signatures overlapping QTL associated to milk traits

| **BTA** | **BP±5 Kb** | **Approach** | **QTL-ID** | **Trait** | **PMID** |
| --- | --- | --- | --- | --- | --- |
| 6 | 88608014 | CLR (g) | 138060 | Milk protein percent | 27760518 |
| 6 | 88613421 | CLR (g) | 138061 | Milk protein percent | 27760518 |
| 6 | 88615096 | CLR (g) | 138062 | Milk protein percent | 27760518 |
| 6 | 88608014 | CLR (g) | 117525 | Milk unglycosylated kappa casein percentage | 27485317 |
| 6 | 88613421 | CLR (g) | 117526 | Milk unglycosylated kappa casein percentage | 27485317 |
| 6 | 88615096 | CLR (g) | 117527 | Milk unglycosylated kappa casein percentage | 27485317 |
| 6 | 88608014 | CLR (g) | 114586 | Milk glycosylated kappa casein percentage | 27485317 |
| 6 | 88613421 | CLR (g) | 114587 | Milk glycosylated kappa casein percentage | 27485317 |
| 6 | 88615096 | CLR (g) | 114588 | Milk glycosylated kappa casein percentage | 27485317 |
| 6 | 88608014 | CLR (g) | 109502 | Milk kappa casein amount | 27485317 |
| 6 | 88613421 | CLR (g) | 109503 | Milk  kappa casein percentage | 27485317 |
| 6 | 88615096 | CLR (g) | 109504 | Milk kappa casein amount | 27485317 |
| 10 | 1.03E+08 | CLR (g) | 34802 | Milk stearic acid content | 25511820 |
| 10 | 1.03E+08 | CLR (g) | 34846 | Milk stearic acid content | 25511820 |
| 11 | 42699221 | CLR (g) | 116066 | Milk glycosylated kappa casein percentage | 27485317 |
| 11 | 42822777 | CLR (g) | 116935 | Milk glycosylated kappa casein percentage | 27485317 |
| 11 | 42955239 | CLR (g) | 112772 | Milk kappa casein amount | 27485317 |
| 11 | 42822777 | CLR (g) | 111716 | Milk kappa casein amount | 27485317 |
| 11 | 42699221 | CLR (g) | 112349 | Milk kappa casein amount | 27485317 |
| 11 | 42949673 | CLR (g) | 112394 | Milk kappa casein amount | 27485317 |
| 11 | 43076962 | CLR (g) | 64158 | Milk riboflavin content | 25771056 |
| 14 | 4042678 | CLR (g) | 103704 | Milk fat percentage | 27287773 |
| 14 | 4048011 | CLR (g) | 103705 | Milk fat percentage | 27287773 |
| 14 | 4042678 | CLR (g) | 102666 | Milk fat yield | 27287773 |
| 14 | 4048011 | CLR (g) | 102667 | Milk fat yield | 27287773 |
| 14 | 4046383 | CLR (g) | 157939 | Milking speed | 29705414 |
| 14 | 4042678 | CLR (g) | 33205 | Milk fat percentage | 25511820 |
| 14 | 4048011 | CLR (g) | 33206 | Milk fat percentage | 25511820 |
| 14 | 7346806 | CLR (g) | 157979 | Milking speed | 29705414 |
| 18 | 41703365 | CLR (g) | 112301 | Milk kappa casein amount | 27485317 |
| 18 | 41701692 | CLR (g) | 112887 | Milk kappa casein amount | 27485317 |
| 20 | 35224329 | CLR (g) | 105315 | Milk protein percent | 27287773 |
| 4 | 72070491 | CLR (t) | 114162 | Milk casein percent | 27485317 |
| *6* | 85083646 | CLR (t) | 117031 | Milk unglycosylated kappa casein percentage | 27485317 |
| 6 | 85082424 | CLR (t) | 117052 | Milk unglycosylated kappa casein percentage | 27485317 |
| 6 | 85086782 | CLR (t) | 117053 | Milk unglycosylated kappa casein percentage | 27485317 |
| 6 | 85082424 | CLR (t) | 114297 | Milk glycosylated kappa casein percentage | 27485317 |
| 6 | 85086782 | CLR (t) | 114298 | Milk glycosylated kappa casein percentage | 27485317 |
| 6 | 85083646 | CLR (t) | 114311 | Milk glycosylated kappa casein percentage | 27485317 |
| 6 | 85083646 | CLR (t) | 109107 | Milk kappa casein amount | 27485317 |
| 6 | 85082424 | CLR (t) | 109127 | Milk kappa casein amount | 27485317 |
| 6 | 85086782 | CLR (t) | 109128 | Milk kappa casein amount | 27485317 |
| 8 | 29865913 | CLR (t) | 36329 | Milk fat percentage | 25148050 |
| 8 | 29865913 | CLR (t) | 14933 | Milk fat percentage | 25148050 |
| 8 | 57313074 | CLR (t) | 26256 | Milk protein yield | 22449276 |
| 11 | 44049642 | CLR (t) | 64162 | Milk riboflavin content | 25771056 |
| 14 | 66735678 | CLR (t) | 105042 | Milk protein percent | 27287773 |
| 14 | 66729813 | CLR (t) | 105045 | Milk protein percent | 27287773 |
| 14 | 66735678 | CLR (t) | 104448 | Milk fat percentage | 27287773 |
| 14 | 66729813 | CLR (t) | 104625 | Milk fat percentage | 27287773 |
| 3 | 93093392 | F_ST_ | 118961 | Milk unglycosylated kappa casein percentage | 27485317 |
| 3 | 93096227 | F_ST_ | 118962 | Milk unglycosylated kappa casein percentage | 27485317 |
| 3 | 93097949 | F_ST_ | 118963 | Milk unglycosylated kappa casein percentage | 27485317 |
| 3 | 93093392 | F_ST_ | 113177 | Milk  kappa casein percentage | 27485317 |
| 3 | 93096227 | F_ST_ | 113178 | Milk  kappa casein percentage | 27485317 |
| 3 | 93097949 | F_ST_ | 113179 | Milk  kappa casein percentage | 27485317 |
| 3 | 13215818 | F_ST_ | 106015 | Milk protein percent | 27287773 |
| 6 | 1.07E+08 | F_ST_ | 118967 | Milk unglycosylated kappa casein percentage | 27485317 |
| 6 | 1.07E+08 | F_ST_ | 113281 | Milk  kappa casein percentage | 27485317 |
| 6 | 94171894 | F_ST_ | 111586 | Milk  kappa casein percentage | 27485317 |
| 6 | 94176758 | F_ST_ | 111587 | Milk  kappa casein percentage | 27485317 |
| 6 | 94177972 | F_ST_ | 111588 | Milk  kappa casein percentage | 27485317 |
| 10 | 12919428 | F_ST_ | 32283 | Milk conjugated linoleic acid content | 24909189 |
| 11 | 44314266 | F_ST_ | 119647 | Milk  alpha s2 casein percentage | 27485317 |
| 11 | 49851472 | F_ST_ | 112585 | Milk  alpha s2 casein percentage | 27485317 |
| 11 | 49856144 | F_ST_ | 112586 | Milk kappa casein percentage | 27485317 |
| 11 | 68103346 | F_ST_ | 113111 | Milk kappa casein percentage | 27485317 |
| 11 | 68103851 | F_ST_ | 113112 | Milk kappa casein percentage | 27485317 |
| 11 | 68104400 | F_ST_ | 113113 | Milk kappa casein percentage | 27485317 |
| 11 | 68104892 | F_ST_ | 113114 | Milk kappa casein percentage | 27485317 |
| 11 | 68105580 | F_ST_ | 113115 | Milk kappa casein percentage | 27485317 |
| 14 | 3692430 | F_ST_ | 103748 | Milk fat percentage | 27287773 |
| 14 | 3692430 | F_ST_ | 102649 | Milk fat yield | 27287773 |
| 14 | 68683085 | F_ST_ | 66156 | Milk yield | 26515756 |
| 14 | 3686405 | F_ST_ | 100210 | Milk fat yield | 27006194 |
| 16 | 26461264 | F_ST_ | 32297 | Milk conjugated linoleic acid content | 24909189 |
| 16 | 26461264 | F_ST_ | 32342 | Milk trans vaccenic acid | 24909189 |
| 25 | 14408320 | F_ST_ | 99597 | Milk fat yield | 27006194 |
| 25 | 14410371 | F_ST_ | 99612 | Milk fat yield | 27006194 |
| 26 | 18401720 | F_ST_ | 34120 | Milk C14 index | 25511820 |
| 26 | 18404628 | F_ST_ | 34121 | Milk C14 index | 25511820 |
| 26 | 18406729 | F_ST_ | 34122 | Milk C14 index | 25511820 |
| 26 | 18407335 | F_ST_ | 34123 | Milk C14 index | 25511820 |
| 26 | 18407930 | F_ST_ | 34124 | Milk C14 index | 25511820 |
| 26 | 49307320 | F_ST_ | 53269 | Milk fat percentage | 21831322 |
| 26 | 49307320 | F_ST_ | 53270 | Milk fat yield | 21831322 |
| 26 | 49307320 | F_ST_ | 53272 | Milk protein percent | 21831322 |
| 26 | 49307320 | F_ST_ | 53273 | Milk protein yield | 21831322 |

**S17** Selection signatures overlapping QTL associated to reproduction traits

| **BTA** | **BP±5 Kb** | **Approach** | **QTL-ID** | **Trait** | **PMID** |
| --- | --- | --- | --- | --- | --- |
| X | 81345114 | CLR (g) | 74840 | scrotal circumference | 23785023 |
| X | 1.05E+08 | CLR (g) | 77096 | scrotal circumference | 23785023 |
| X | 1.05E+08 | CLR (g) | 80347 | scrotal circumference | 23785023 |
| X | 81348511 | CLR (g) | 72270 | scrotal circumference | 23785023 |
| X | 81343883 | CLR (g) | 81220 | scrotal circumference | 23785023 |
| X | 81348511 | CLR (g) | 83571 | percentage normal sperm | 23785023 |
| X | 81348511 | CLR (g) | 85758 | age at puberty | 23785023 |
| X | 81345114 | CLR (g) | 87802 | age at puberty | 23785023 |
| X | 1.05E+08 | CLR (g) | 93086 | age at puberty | 23785023 |
| X | 1.05E+08 | CLR (g) | 94755 | age at puberty | 23785023 |
| X | 81342571 | CLR (g) | 94241 | age at puberty | 23785023 |
| 1 | 1.04E+08 | CLR (g) | 56271 | sperm motility | 26051317 |
| 2 | 1516011 | CLR (g) | 29776 | age at puberty | 22100599 |
| 11 | 43076962 | CLR (g) | 125299 | twinning | 19220232 |
| X | 81335851 | F_ST_ | 74838 | scrotal circumference | 23785023 |
| X | 81331743 | F_ST_ | 75247 | scrotal circumference | 23785023 |
| X | 81334127 | F_ST_ | 78488 | scrotal circumference | 23785023 |
| X | 81327217 | F_ST_ | 80087 | scrotal circumference | 23785023 |
| X | 81335851 | F_ST_ | 87800 | age at puberty | 23785023 |
| X | 81331743 | F_ST_ | 88068 | age at puberty | 23785023 |
| X | 81334127 | F_ST_ | 92295 | age at puberty | 23785023 |
| X | 81327217 | F_ST_ | 93779 | age at puberty | 23785023 |
| X | 81332724 | F_ST_ | 94240 | age at puberty | 23785023 |
| X | 81328513 | F_ST_ | 83570 | percentage normal sperm | 23785023 |
| X | 81328513 | F_ST_ | 85757 | age at puberty | 23785023 |
| X | 81328513 | F_ST_ | 72269 | scrotal circumference | 23785023 |
| 25 | 1453757 | F_ST_ | 143251 | interval to first estrus after calving | 29178833 |
| 25 | 1454235 | F_ST_ | 143254 | interval to first estrus after calving | 29178833 |
| 25 | 1454540 | F_ST_ | 143255 | interval to first estrus after calving | 29178833 |
| 25 | 1454400 | F_ST_ | 143256 | interval to first estrus after calving | 29178833 |
| 25 | 1455516 | F_ST_ | 143258 | interval to first estrus after calving | 29178833 |
| 25 | 1456582 | F_ST_ | 143259 | interval to first estrus after calving | 29178833 |
| 25 | 1459049 | F_ST_ | 143263 | interval to first estrus after calving | 29178833 |
| 25 | 1458076 | F_ST_ | 143268 | interval to first estrus after calving | 29178833 |
| 25 | 1459437 | F_ST_ | 143280 | interval to first estrus after calving | 29178833 |
| 25 | 1460228 | F_ST_ | 143284 | interval to first estrus after calving | 29178833 |
| 25 | 1460345 | F_ST_ | 143290 | interval to first estrus after calving | 29178833 |
| 25 | 1460383 | F_ST_ | 143294 | interval to first estrus after calving | 29178833 |
| 25 | 1460516 | F_ST_ | 143298 | interval to first estrus after calving | 29178833 |
| 25 | 1460922 | F_ST_ | 143302 | interval to first estrus after calving | 29178833 |
| 25 | 1457717 | F_ST_ | 143306 | interval to first estrus after calving | 29178833 |
| 25 | 1452675 | F_ST_ | 143405 | interval to first estrus after calving | 29178833 |
| 25 | 1451810 | F_ST_ | 143406 | interval to first estrus after calving | 29178833 |
| 25 | 1451646 | F_ST_ | 143407 | interval to first estrus after calving | 29178833 |
| 25 | 1454235 | F_ST_ | 142875 | Interval from first to last insemination | 29178833 |
| 25 | 1454400 | F_ST_ | 142876 | Interval from first to last insemination | 29178833 |
| 25 | 1454540 | F_ST_ | 142877 | Interval from first to last insemination | 29178833 |
| 25 | 1455516 | F_ST_ | 142879 | Interval from first to last insemination | 29178833 |
| 25 | 1452675 | F_ST_ | 142880 | Interval from first to last insemination | 29178833 |
| 25 | 1456582 | F_ST_ | 142881 | Interval from first to last insemination | 29178833 |
| 25 | 1457717 | F_ST_ | 142882 | Interval from first to last insemination | 29178833 |
| 25 | 1458076 | F_ST_ | 142883 | Interval from first to last insemination | 29178833 |
| 25 | 1459437 | F_ST_ | 142884 | Interval from first to last insemination | 29178833 |
| 25 | 1459049 | F_ST_ | 142885 | Interval from first to last insemination | 29178833 |
| 25 | 1460228 | F_ST_ | 142888 | Interval from first to last insemination | 29178833 |
| 25 | 1460345 | F_ST_ | 142890 | Interval from first to last insemination | 29178833 |
| 25 | 1460383 | F_ST_ | 142892 | Interval from first to last insemination | 29178833 |
| 25 | 1460516 | F_ST_ | 142894 | Interval from first to last insemination | 29178833 |
| 25 | 1460922 | F_ST_ | 142901 | Interval from first to last insemination | 29178833 |
| 25 | 1451810 | F_ST_ | 142949 | Interval from first to last insemination | 29178833 |
| 25 | 1451646 | F_ST_ | 142950 | Interval from first to last insemination | 29178833 |
| 25 | 1459813 | F_ST_ | 143076 | Interval from first to last insemination | 29178833 |
| 25 | 1460478 | F_ST_ | 143077 | Interval from first to last insemination | 29178833 |
| 25 | 1459801 | F_ST_ | 143078 | Interval from first to last insemination | 29178833 |
| 25 | 1459805 | F_ST_ | 143079 | calving ease | 29178833 |
| 25 | 1460500 | F_ST_ | 143080 | calving ease | 29178833 |
| 25 | 1459747 | F_ST_ | 143082 | calving ease | 29178833 |
| 25 | 1458546 | F_ST_ | 143084 | calving ease | 29178833 |
| 25 | 1460456 | F_ST_ | 143085 | calving ease | 29178833 |
| 25 | 1459084 | F_ST_ | 143086 | calving ease | 29178833 |
| 25 | 1458088 | F_ST_ | 143087 | calving ease | 29178833 |
| 25 | 1456747 | F_ST_ | 143088 | calving ease | 29178833 |
| 25 | 1457016 | F_ST_ | 143090 | calving ease | 29178833 |
| 25 | 1457717 | F_ST_ | 145578 | calving ease | 29178833 |
| 25 | 1454400 | F_ST_ | 145579 | calving ease | 29178833 |
| 25 | 1454235 | F_ST_ | 145580 | calving ease | 29178833 |
| 25 | 1454540 | F_ST_ | 145582 | calving ease | 29178833 |
| 25 | 1455516 | F_ST_ | 145584 | calving ease | 29178833 |
| 25 | 1456582 | F_ST_ | 145589 | calving ease | 29178833 |
| 25 | 1458076 | F_ST_ | 145612 | calving ease | 29178833 |
| 25 | 1459049 | F_ST_ | 145662 | calving ease | 29178833 |
| 25 | 1459437 | F_ST_ | 145664 | calving ease | 29178833 |
| 25 | 1460922 | F_ST_ | 145665 | calving ease | 29178833 |
| 25 | 1460516 | F_ST_ | 145669 | calving ease | 29178833 |
| 25 | 1453757 | F_ST_ | 145679 | interval from first to last insemination | 29178833 |
| 25 | 1460383 | F_ST_ | 145681 | interval from first to last insemination | 29178833 |
| 25 | 1460228 | F_ST_ | 145683 | interval to first estrus after calving | 29178833 |
| 25 | 1460345 | F_ST_ | 145685 | calving ease | 29178833 |
| 25 | 1452675 | F_ST_ | 145736 | calving ease | 29178833 |
| 25 | 1451810 | F_ST_ | 145741 | calving ease | 29178833 |
| 25 | 1451646 | F_ST_ | 145742 | calving ease | 29178833 |
| 25 | 1456747 | F_ST_ | 145768 | calving ease | 29178833 |
| 25 | 1457016 | F_ST_ | 145769 | calving ease | 29178833 |
| 25 | 1453757 | F_ST_ | 143671 | calving ease | 29178833 |
| 25 | 1460468 | F_ST_ | 142889 | calving ease | 29178833 |
| 25 | 1460468 | F_ST_ | 143292 | calving ease | 29178833 |
| 25 | 1460468 | F_ST_ | 145675 | calving ease | 29178833 |
| 26 | 49307320 | F_ST_ | 53268 | calving ease | 29178833 |
| 26 | 49307320 | F_ST_ | 53275 | calving ease | 29178833 |

**S18** Selection signatures overlapping QTLs associated to health traits

| **BTA** | **BP±5 Kb** | **Approach** | **QTL-ID** | **Trait** | **PMID** |
| --- | --- | --- | --- | --- | --- |
| 1 | 118638525 | CLR (g) | 16300 | Johne’s disease tolerance | 20477805 |
| 23 | 29734356 | CLR (t) | 153949 | Bovine leukemia virus susceptibility | 29439661 |
| 10 | 66551708 | F_ST_ | 160141 | bovine respiratory disease susceptibility | 30229962 |

**S19** Selection signatures overlapping QTL having association with growth traits

| **BTA** | **BP±5 Kb** | **Approach** | **QTL-ID** | **Trait** | **PMID** |
| --- | --- | --- | --- | --- | --- |
| 1 | 104914011 | CLR (g) | 130766 | dry matter intake | 28521758 |
| 1 | 118638525 | CLR (g) | 66303 | body weight (yearling) | 19966163 |
| 1 | 118638525 | CLR (g) | 66757 | body weight (yearling) | 19966163 |
| 1 | 118638525 | CLR (g) | 66758 | body weight gain | 19966163 |
| 1 | 118638525 | CLR (g) | 66759 | body weight gain | 19966163 |
| 1 | 118638525 | CLR (g) | 66760 | body weight gain | 19966163 |
| 5 | 110751512 | CLR (g) | 67076 | body weight (weaning) | 19966163 |
| 5 | 110751512 | CLR (g) | 67077 | body weight (yearling) | 19966163 |
| 5 | 110751512 | CLR (g) | 67078 | body weight gain | 19966163 |
| 14 | 80695704 | CLR (g) | 68644 | body weight (yearling) | 19966163 |
| 15 | 43188647 | CLR (g) | 21072 | residual feed intake | 22497295 |
| 8 | 59626198 | CLR (t) | 67948 | body weight gain | 19966163 |
| 6 | 99188303 | F_ST_ | 67629 | body weight gain | 19966163 |
| 26 | 49307320 | F_ST_ | 53271 | net merit | 21831322 |

**S20** Selection signatures overlapping QTL associated to carcass traits

| **BTA** | **BP±5 Kb** | **Approach** | **QTL-ID** | **Trait** | **PMID** |
| --- | --- | --- | --- | --- | --- |
| 8 | 29865913 | CLR (t) | 36650 | sub cutaneous fat | 25273628 |
| 21 | 40464721 | CLR (t) | 137165 | shear force | 28727016 |
| 21 | 40343860 | CLR (t) | 137168 | shear force | 28727016 |
| 2 | 104318604 | F_st_ | 151795 | muscle iron content | 29163638 |
| 8 | 53819922 | F_st_ | 151527 | muscle anserine content | 29163638 |

**S21** Selection signatures overlapping QTLs associated to exterior traits

| **BTA** | **BP±5 Kb** | **Approach** | **QTL-ID** | **Trait** | **PMID** |
| --- | --- | --- | --- | --- | --- |
| 5 | 12396649 | CLR (t) | 147130 | teat length | 29115939 |
| 5 | 12397321 | CLR (t) | 147131 | teat length | 29115939 |
| 11 | 4284312 | CLR (t) | 154169 | stature | 29459679 |
| 11 | 4284312 | CLR (t) | 154305 | stature | 29459679 |
| 11 | 4284312 | CLR (t) | 154306 | stature | 29459679 |
| 14 | 68683085 | F_ST_ | 66138 | Duration of inactivity during novel object test | 26515756 |
| 25 | 1455431 | F_ST_ | 22739 | Stature | 23031427 |
| 26 | 49307320 | F_ST_ | 53274 | rump angle | 21831322 |

**S22** Selection signatures that overlapped with QTL terms

| **Trait** | **Gir** | **Tharparkar** | **F_ST_** |
| --- | --- | --- | --- |
| Milk | 32 | 18 | 38 |
| Reproduction | 14 | 11 | 85 |
| Growth | 11 | 1 | 2 |
| Exterior | - | 5 | 3 |
| Health | 1 | 1 | 1 |
| Meat | - | 3 | 2 |

**S23** PANTHER pathways in Gir cattle

| **.No.** | **PANTHER Pathways** | **Genes** |
| --- | --- | --- |
| 1. | 5HT2 type receptor mediated signaling pathway (P04374) | *GNA14, CACNA1D* |
| 2. | Alzheimer disease-amyloid secretase pathway (P00003) | *MAPK4, CACNA1D* |
| 3. | Alpha adrenergic receptor signaling pathway (P00002) | *ITPR1* |
| 4. | Integrin signalling pathway (P00034) | *MAP3K3, COL13A1, COL4A1* |
| 5. | Inflammation mediated by chemokine and cytokine signaling pathway (P00031) | *ITPR1, GNA14* |
| 6. | Huntington disease (P00029) | *GRIK5* |
| 7. | Heterotrimeric G-protein signaling pathway-Gq alpha and Go alpha mediated pathway (P00027) | *CACNA1B, ITPR1, GNA14* |
| 8. | Wnt signaling pathway (P00057) | *GNA14, CDH13, ITPR1, HLTF* |
| 9. | Thyrotropin-releasing hormone receptor signaling pathway (P04394) | *CACNA1B, GNA14* |
| 10. | Ras Pathway (P04393) | *PDPK1, RPS6KA2, TIAM1, PAK2* |
| 11. | T cell activation (P00053) | *ITPR1* |
| 12. | FGF signaling pathway (P00021) | *MAP3K3* |
| 13. | TGF-beta signaling pathway (P00052) | *TGFBR1, TGFB2, BMPR1A, BMP5* |
| 14. | Oxytocin receptor mediated signaling pathway (P04391) | *PLCB1, SNAP25, PRKCE* |
| 15. | EGF receptor signaling pathway (P00018) | *MAP3K3* |
| 16. | PI3 kinase pathway (P00048) | *GNA14* |
| 17. | PDGF signaling pathway (P00047) | *ITPR1,RPS6KA5* |
| 18. | Cadherin signaling pathway (P00012) | *CDH13* |
| 19. | Muscarinic acetylcholine receptor 1 and 3 signaling pathway (P00042) | *ITPR1, GNA14* |
| 20. | Angiotensin II-stimulated signaling through G proteins and beta-arrestin (P05911) | *ITPR1* |
| 21. | Metabotropic glutamate receptor group I pathway (P00041) | *CACNA1B, GRIK5* |
| 22. | Cortocotropin releasing factor receptor signaling pathway (P04380) | *GNA14* |
| 23. | CCKR signaling map (P06959) | *ITPR1* |
| 24. | Gonadotropin-releasing hormone receptor pathway (P06664) | *MAP3K3, ITPR1, CACNA1D* |
| 25. | De novo purine biosynthesis | *GARS, ASL* |
| 26. | Nicotinic acetylcholine receptor signaling pathway (P00044) | *MYO5B, CACNA1D* |
| 27. | GABA-B receptor II signaling (P05731) | *CACNA1B* |
| 28. | Endogenous cannabinoid signaling (P05730) | *CACNA1B* |
| 29. | Metabotropic glutamate receptor group III pathway (P00039) | *CACNA1B, SNAP25, VTI1A, GRIA2* |
| 30. | Ionotropic glutamate receptor pathway (P00037) | *CACNA1B, GRIK5* |
| 31. | Insulin/IGF pathway-protein kinase B signaling cascade (P00033) |  |
|  | Insulin/IGF pathway-mitogen activated protein kinase kinase/MAP kinase cascade (P00032) | *RPS6KA5* |
| 32. | Arginine Biosynthesis (P02728) | *ASL* |
| 33. | Histamine H1 receptor mediated signaling pathway (P04385) | *ITPR1, GNA14* |
| 34. | Endothelin signaling pathway (P00019) | *ITPR1, GNA14* |
| 35. | Ubiquitin proteasome pathway (P00060) | *UBA2* |
| 36. | Metabotropic glutamate receptor group I pathway (P00041) | *GRIK5, ITPR1* |
| 37. | p38 MAPK pathway (P05918) | *RPS6KA5* |
| 38. | Beta2 adrenergic receptor signaling pathway (P04378) | *CACNA1D* |
| 39. | Beta1 adrenergic receptor signaling pathway (P04377) | *CACNA1D* |
| 40. | B cell activation (P00010) | *MAP3K3, ITPR1* |
| 41. | Endogenous cannabinoid signaling (P05730) | *CACNA1B* |
| 42. | Endothelin signaling pathway (P00019) | *ITPR1, GNA14* |
| 43. | Metabotropic glutamate receptor group IIpathway (P00040) | *CACNA1B* |
| 44. | Oxytocin receptor mediated signaling pathway (P04391) | *GNA14, CACNA1D* |

**S24** Panther pathways in Tharparkar cattle

| **S.No.** | **PANTHER Pathways** | **Genes** |
| --- | --- | --- |
| 1. | Inflammation mediated by chemokine and cytokine signaling pathway (P00031) | *XCR1* |
| 2. | Wnt signaling pathway (P00057) | CTNNA3, SMARCC1, TLE1, CSNK1G2 |
| 3. | Ras Pathway (P04393) | RPS6KA2 |
| 4. | Synaptic vesicle trafficking(P05734) | *UNC13B* |
| 5. | Cadherin signaling pathway (P00012) | *CTNNA3* |
| 6. | CCKR signaling map (P06959) | *PRKD1* |
| 7. | Axon guidance mediated by semaphorins (P00007) | *SEMA3A* |
| 8. | VEGF signaling pathway (P00056) | *PRKD1* |
| 9. | PDGF signaling pathway (P00047) | *RPS6KA2, SRGAP3* |
| 10. | Interleukin signaling pathway (P00036) | *RPS6KA2* |
| 11. | Interferon-gamma signaling pathway (P00035) | SOCS7 |
| 12. | Parkinson Disease (P00059) | CSNK1G2 |
| 13. | Xanthine and guanine salvage pathway (P02788) | GDA |
| 14. | Insulin/IGF pathway-protein kinase B signaling cascade (P00033) | *RPS6KA2* |
| 15. | Insulin/IGF pathway-mitogen activated protein kinase kinase/MAP kinase cascade (P00032) |  |

**S25** Panther pathways of genes identified by F_ST_ approach

| **S.No** | **PANTHER Pathways** | **Between breeds** |
| --- | --- | --- |
| 1. | Axon guidance mediated by Slit/Robo (P00008) | *ROBO1, SLIT3, SLIT2* |
| 2. | Apoptosis signaling pathway (P00006) | *MAPK10, APAF1, M6PR, PRKCE, BCL2L1, APAF1, MAP3K14* |
| 3. | 5HT4 type receptor mediated signaling pathway | *KCNK3, KCNK9* |
| 4. | Angiogenesis (P00005) | *RBPJ, MARK10* |
| 5. | 5HT3 type receptor mediated signaling pathway (P04375) | *KCNK3, KCNK9* |
| 6. | Alzheimer disease-presenilin pathway (P00004) | *RBPJ, TCF7L1, ERN1* |
| 7. | Interleukin signaling pathway (P00036) | *IL12RB1* |
| 8. | 5HT2 type receptor mediated signaling pathway (P04374) | *KCNK3, GNAQ, PLCB4, KCNK9* |
| 9. | Alzheimer disease-amyloid secretase pathway (P00003) | *MAPK10* |
| 10. | Interferon-gamma signaling pathway (P00035) | *MAPK10* |
| 11. | 5HT1 type receptor mediated signaling pathway (P04373) | *KCNK3, KCNK9* |
| 12. | Alpha adrenergic receptor signaling pathway (P00002) | *PLCB4* |
| 13. | Integrin signalling pathway (P00034) | *MAPK10* |
| 14. | Inflammation mediated by chemokine and cytokine signaling pathway (P00031) | *GNAQ, PLCB4* |
| 15. | Nicotine pharmacodynamics pathway (P06587) | *KCNK3, KCNK9* |
| 16. | Huntington disease (P00029) | *DNAH7, VAT1L, NCOR2, DNAH8, HAP1, KALRN, AKT3, APAF1* |
| 17. | Heterotrimeric G-protein signaling pathway-Gq alpha and Go alpha mediated pathway (P00027) | *GNAQ, PLCB4* |
| 18. | Wnt signaling pathway (P00057) | *GNAQ, PLCB4, TNF7L1* |
| 19. | Thyrotropin-releasing hormone receptor signaling pathway (P04394) | *GNAQ, PLCB4* |
| 20. | Toll receptor signaling pathway (P00054) | *MAPK1O* |
| 21. | Ras Pathway (P04393) | *MAPK1O* |
| 22. | T cell activation (P00053) | *MAPK1O* |
| 23. | FGF signaling pathway (P00021) | *MAPK1O* |
| 24. | TGF-beta signaling pathway (P00052) | *MAPK1O, TGFB2* |
| 25. | Oxytocin receptor mediated signaling pathway (P04391) | *GNAQ, PLCB4* |
| 26. | FAS signaling pathway (P00020) | *MAPK10, APAF1* |
| 27. | Plasminogen activating cascade (P00050) | *PLG* |
| 28. | EGF receptor signaling pathway (P00018) | *MAPK10, PHLBD2* |
| 29. | Parkinson disease (P00049) | *MAPK10* |
| 30. | PI3 kinase pathway (P00048) | *GNAQ* |
| 31. | Opioid proopiomelanocortin pathway (P05917) | *KCNK3, KCNK9* |
| 32. | PDGF signaling pathway (P00047) | *MAPK10* |
| 33. | Oxidative stress response (P00046) | *MAPK10* |
| 34. | Opioid proenkephalin pathway (P05915) | *KCNK3, KCNK9* |
| 35. | Histamine H1 receptor mediated signaling pathway (P04385) | *GNAQ, PLCB4* |
| 36. | Notch signaling pathway (P00045) | *RBPJ* |
| 37. | Nicotine degradation (P05914) | *FMO3* |
| 38. | Cadherin signaling pathway (P00012) | *TCF7L1* |
| 39. | Blood coagulation (P00011) | *PLG* |
| 40. | Dopamine receptor mediated signaling pathway (P05912) | *KCNK3, KCNK9* |
| 41. | B cell activation (P00010) | *MAPK10* |
| 42. | Muscarinic acetylcholine receptor 1 and 3 signaling pathway (P00042) | *GNAQ, PLCB4* |
| 43. | Angiotensin II-stimulated signaling through G proteins and beta-arrestin (P05911) | *GNAQ* |
| 44. | Metabotropic glutamate receptor group I pathway (P00041) | *GNA11* |
| 45. | Cortocotropin releasing factor receptor signaling pathway (P04380) | *GNAQ* |
| 46. | CCKR signaling map (P06959) | *MAPK10, MAP3K14* |
| 47. | Gonadotropin-releasing hormone receptor pathway (P06664) | *GNAQ, TGFB2, TCF7L1, MAP3K14* |
| 48. | Oxidative stress response (P00046) | *MAPK10* |
| 49. | Vitamin D metabolism and pathway (P04396) | *FDX2* |
| 50. | p53 pathway (P00059) | *APAF1* |

**S26** Gene ontology: Cellular component

| **Process** | **GO term** | **Gir** | **Tharparkar** | **F_ST_** |
| --- | --- | --- | --- | --- |
| **Biological adhesion** | **22610** | *COL13A1* | *SPON1* | *PLXNA4, NCAM1,IL12RB1, ICAM5, ATRN* |
| **Biological regulation** | **65007** | *BBX, CACNA1B, RAB20, WWTR1, FYB1, GRIK5, BCL11A, PARG, GRID2, CP, ITPR1, LDB2, CELF4, GCFC2, GNA14, MAPK4, ABRACL, TBC1D22A, PARG, TLE1* | *KIAA1614, BATF3, SP4, PKIB, PDE2A, CNOT1, VTCN1, GCFC2, PRKD1, STIM2, UNC13B, EYA2, PDE2A, SEMA3A, SMARCC1, NDRG4, XCR1, CSNK1G2, SRGAP3* | *PLXNA4, IL12RB1, RBPJ, NOTO, MEGF11, MAPK10, IL1F10, KCNK3, SCG5, GNAQ, ERN1, CDK13, TGFB2, TCF7L1, PHLDB2, GRID1, PLCB4, GPC3, KCNK9, SULF1, FMNL2, GRB10* |
| **Cell population proliferation** | **8283** | *-* | *-* | *1L12RB1, TGFB2* |
| **Cellular component organization or biogenesis** | **71840** | *CACNA1B, COL13A1, MAP4, PARG, MUM1, CELF4, RPL3, PARG, SEC31A, SMYD3* | *KIAA1614, TRAK1, IFT81, PDE2A, MAP4, MUM1, UNC13B, EYA2, MAP4, PDE2A, VCP, SEMA3A, SMARCC1, LCP1* | *PLXNA4, NCAM1, SLIT1, SLIT3, SEPT10, PHLDB2, PDS5A, SULF1, FMNL2, ATRN* |
| **Cellular process** | **9987** | *BBX, CACNA1B, RAB20, COL13A1, WWTR1, FYB1, GRIK5, MAP4, ASL, BCL11A, PARG, GRID2, ITPR1, LDB2, MUM1, UBA2, CELF4, GCFC2, GNA14, GARS, VKORC1L1, GUSB, MAPK4, ABRACL, MAP4, ASL, RPL3, XPR1, MARCH3, FST L4, PARG, CRCP, PUS10, SEC31A, OGDHL, SMYD3* | *SETD6, KIAA1614, TRAK1, PIGO, IFT81, BATF3, SP4, PKIB, PDE2A, MAP4, CNOT1, MUM1, VTCN1, GCFC2, PRKD1, STIM2, UNC13B, FANCG, EYA2, MAP4, THUMPD3, PDE2A, TUSC3, VCP, SEMA3A, SMARCC1, NDRG4, XCR1, FST L4, SETD6, CSNK1G2, SRGAP3, TLE1, LCP1* | *PLXNA4, NCAM1, WLS, IL12RB1, SLIT1, RBPJ, NOTO, MEGF11, MAPK10, IL1F10, MANIA2, SCG5, ELOVL5, GNAQ, ERN1, SLIT3, CDK13, TGFB2, SEPT10, TCF7L1, PHLDB2, GRID1, PLCB4, PDS5A, GPC3, SULF1, FMNL2, GRB10, ATRN, KIF6* |
| **Developmental process** | **32502** | *COL13A1, MAP4, BCL11A, GFRA1, LDB2, MAP4, FST L4* | *TRAK1, MAP4, UNC13B, EYA2, MAP4, SEMA3A, FST L4* | *PLXNA4, NCAM1, SLIT1, NOTO, SLIT3, SULF1, ATRN, FMNL2* |
| **Immune system process** | **2376** | *FYB1* | *VTCN1, XCR1* | *IL12RB1, IL1F10* |
| **localization** | **51179** | *CACNA1B, RAB20, IPO7, SLC6A14, FYB1, CP, ITPR1, TBC1D22A, XPR1, CANA1D, SEC31A* | *KIAA1614, SCAMP4, TRAK1, IFT81, STIM2, UNC13B, RANBP17, KCNQ4, VCP, SEMA3A, XCR1, SRGAP3* | *PLXNA4, WLS, KCNK3, ABCC1, SCG5, SEPT10, SORCS2, PLCB4, GPC3, KCNK9, FMNL2, ATRN, SLC7A1* |
| **locomotion** | **40011** | *-* | *SEMA3A, XCR1, SRGAP3* | *PLXNA4, NCAM1, SLIT1, SLIT3, GPC3, FMNL2, ATRN* |
| **Metabolic process** | **8152** | *BBX, WWTR1, ASL, BCL11A, PARG, LDB2, MUM1, UBA2, CELF4, GCFC2, GARS, VKORC1L1, GUSB, MAPK4, ASL, GARS, MARCH3, PARG, CRCP, PUS10, OGDHL, SMYD3* | *SETD6, PIGO, BATF3, SP4, PKIB, PDE2A, CNOT1, MUM1, GCFC2, EYA2, THUMPD3, PDE2A, TUSC3, VCP, SMARCC1, SETD6, CSNK1G2* | *IL12RB1, RBPJ, NOTO, CAPN5, MAPK10, IL1F10, MAN1A2, ELOVL5, CDK13, TGFB2, TCF7L1, PDS5A, GALM, SULF1* |
| **Multi-organism process** | **51704** | *-* | *-* | *IL1F10* |
| **Multi-cellular organismal process** | **32501** | *CACNA1B, COL13A1, GFRA1, LDB2, MAP4, FST L4, BCL11A* | *TRAK1, MAP4, VTCN1, SEMA3A, FST L4* | *PLXNA4, NCAM1, SLIT1, NOTO, SLIT3, SULF1, ATRN* |
| **Response to stimuli** | **50896** | *RAB20, WWTR1, FYB1, PARG, MUM1, GNA14, MAPK4, XPR1, PARG* | *PDE2A, MUM1, VTCN1, PRKD1, FANCG, EYA2, PDE2A, VCP, SEMA3A, NDRG4, XCR1, CSNK11G2, TLE1* | *PLXNA4, NCAM1, IL12RB1, SLIT1, RBPJ, MEGF11, MAPK10, IL1F10, GNAQ, ERN1, SLIT3, TGFB2, TCF7L1, PLCB4, PDS5A, GPC3, SULF1, GRB10, SLC7A1* |
| **Signaling** | **23052** | *CACNA1B, RAB20, WWTR1, FYB1, GRIK5, GRID2, GNA14, MAPK4* | *PDE32A, VTCN1, PRKD1, UNC13B, EYA2, PDE2A, SEMA3A, NDRG4, XCR1, CSNK1G2, TLE1* | *PLXNA4, WLS, IL12RB1, RBPJ, MEGF11, MAPK10, IL1F10, SCG5, GNAQ, ERN1, TGFB2, TCF7L1, GRID1, PLCB4, GPC3, SULF1, GRB10* |

**S27** Gene ontology: Molecular function

| **Molecular function** | **GO** | **Gir** | **Tharparkar** | **F_ST_** |
| --- | --- | --- | --- | --- |
| **Binding** | **5488** | *IL12B, COL13A1, GRIK5, MAP4, GRID2, ITPR1, LDB2, IL12B, MUM1, BCL11A, RPL3, GHRHR, CELF4, ARID3A,  GCFC2, GNA14, WDR44, MAP4,  TBC1D22A, XPR1* | *KIAA1614, TRAK1, IFT81, BATF3, PKIB, PDE2A, MAP4, MUM1, VTCN1, GCFC2, STIM2, UNC13B, OSBPL3, MAP4, KCNQ4, PDE2A, SEMA3A, SMARCC1, XCR1, SRGAP3, AFF4, TLE1, LCP1, VCP* | *NCAM1, WLS, IL12RB1, SLIT1, RBMS3, RBPJ, OSBPL10, NOTO, IL1F10, GNAQ, ERN1, SLIT3, CDK13, ICAM5, TGFB2, SEPT10, TCF7L1, GRID1, SULF1, FMNL2, GRB10, KIF6* |
| **Catalytic activity** | **3824** | *RAB20, ASL, PARG, CP, MINDY4, UBA2, GNA14, GARS, MAPK4, ASL, CTDSPL2, VKORC1L1, MARCH3, TBC1D22A, CRCP, PUS10, OGD, SMYD3* | *SETD6, UGT2A1, PKIB, PDE2A, PDE7A, RPS6KA2, PIGO, EYA2, THUMPD3, PDE2A, SETD6, CSNK1G2* | *FMO3, TNNI1K, INPP4B, CAPN5, MAPK10, MAN1A2, ABCC1,SCG5, ELOVL5, GNAQ, ERN1, HS3ST1, CDK13, SEPT10, PPM1L, PLCB4, GALM, PLG, SULF1, KIF6* |
| **Molecular function regulator** | **98772** | *TBC1D22A, GHRHR* | *PKIB, STIM2, SEMA3A* | *IL1F10, SCG5, TGFB2* |
| **Molecular transducer activity** | **60089** | *IL12B, GRIK5, GRID2, GFRA1, GPR141* | *PKIB, XCR1* | *PLXNA4, IL12RB1, CDK13,GRID1* |
| **Transcription regulator activity** | **140110** | *WWTR1, ARID3A, BCL11A* | *BATF3, SP4, AFF4, TLE1* | *NOTO* |
| **Transporter activity** | **5215** | *CACNA1B, GRIK5, GRID2, ITPR1, XPR1, CACNA1D* | *P2RX7, STIM2, OSBPL3, RANBP17, KCNQ4* | *OSBPL10, KCNK3, ABCC1, GRID1, KCNK9, SLC7A1* |
